# Phototherapy-responsive nanoplatform with hydrogel delivery potentiates immunotherapy and inhibits lung metastasis in osteosarcoma via synergistic glycolysis inhibition and vascular normalization

**DOI:** 10.1186/s12951-026-04906-0

**Published:** 2026-08-12

**Authors:** Mengchao Dong, Tingting Zhang, Yichun Huang, Meng Liu, Yuhui Yuan, Bowen Liu, Qiang Ma, Haotian Zhang, Canhua Huang, Chengcheng Shi, Yi Zhang

**Affiliations:** 1 Department of Orthopedic Surgery, The First Affiliated Hospital of Zhengzhou University, Zhengzhou, 450052 China; 2 Department of Biotherapy Cancer Center and State Key Laboratory of Biotherapy West China Hospital and West China School of Basic Medical Sciences and Forensic Medicine, Sichuan University, Chengdu, 610041 China; 3 The First Affiliated Hospital of Kunming Medical University, Kunming, 650031 China; 4 Department of Pharmacy, The First Affiliated Hospital of Zhengzhou University, 450052 Zhengzhou, China

**Keywords:** pH-responsive hydrogel, Immune modulation, Tumor microenvironment remodeling, Metabolic regulation, Vascular normalization

## Abstract

The immunosuppressive tumor microenvironment (ITME)—marked by acidosis and hypoxia—persists as a critical barrier to cancer immunotherapy, especially for osteosarcoma. Herein, an injectable pH-responsive nanocomposite hydrogel (OP@NPs) is presented to overcome this challenge via coordinated metabolic modulation, vascular normalization, and immune reprogramming. This platform co-delivers a novel lactate metabolism inhibitor (D780, a derivative conjugated by IR780 and diclofenac), Zn²⁺, and the anti-angiogenic agent nintedanib (NIB). D780 inhibits key glycolytic enzymes to reduce lactate production, while exerting photothermal/photodynamic effects that directly ablate tumors and mitigate hypoxia. Through metal coordination, Zn²⁺ assembles NIB and D780 into NPs. It also blocks GLUT1, boosting D780’s inhibition of glycolysis. NIB can restore abnormal vascular structure, thereby alleviating oxidative hypoxia and blocking epithelial-mesenchymal transition. In murine osteosarcoma models, the combined therapy centered on OP@NPs remodelled the tumor immune microenvironment (TME). This transformation led to an accumulation of cytotoxic T cells, dendritic cells and M1 macrophages. The treatment achieved favorable outcomes in inhibiting primary tumor growth and pulmonary metastases. Overall, this work presents a localized targeting strategy to regulate key signaling pathways in osteosarcoma.

**Graphical Abstract:**

**Figure:**
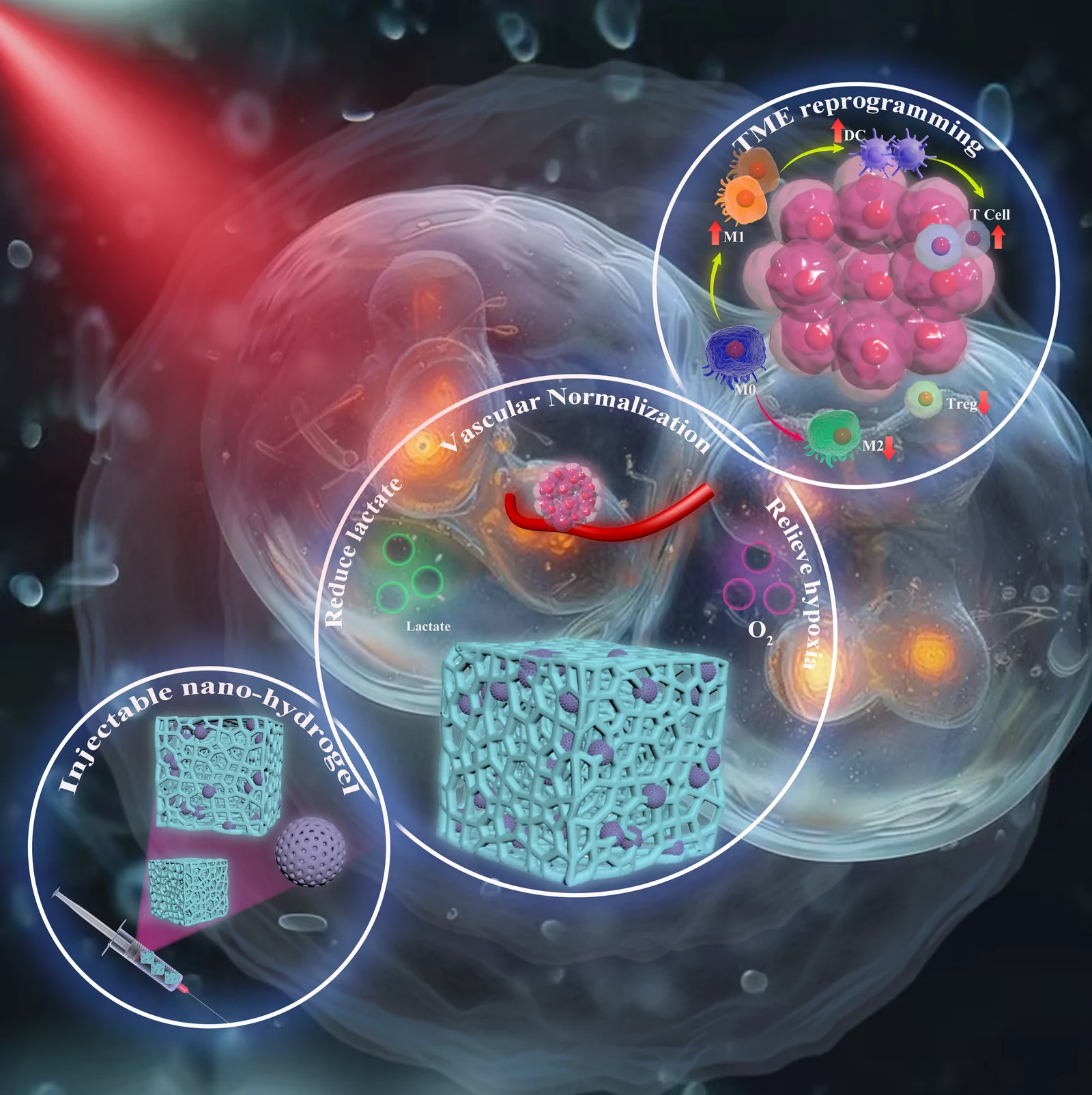

**Supplementary Information:**

The online version contains supplementary material available at https://doi.org/10.1186/s12951-026-04906-0.

## Introduction

Osteosarcoma is an aggressive cancer with strong lung metastatic potential. Recurrence and metastasis typically result in unfavorable survival outcomes [1]. Combined use of chemotherapy, surgery and adjuvant treatment has optimized therapeutic effects, yet recurrence and metastasis remain major causes of unsatisfactory survival [2]. In recent years, immunotherapy has emerged as a highly potent and promising treatment strategy. Nevertheless, the ITME greatly hinders its clinical therapeutic efficacy [3]. The ITME of osteosarcoma typically presents hypoxia, acidosis, and abnormal vasculature. These pathological traits collectively drive tumor immune evasion [4, 5]. Hypoxic conditions and lactic acid buildup impair the activity of functional immune cells. Meanwhile, structurally disordered tumor blood vessels block immune cell penetration and hinder local drug accumulation [6]. Although immune checkpoint inhibitors (such as PD-1/PD-L1 blockade) have shown clinical success in several cancers, these microenvironmental obstacles greatly weaken their clinical performance in osteosarcoma treatment [7, 8]. Therefore, it is critical to develop multifunctional interventions capable of remodeling tumor metabolism, abnormal vasculature, and ITME simultaneously. Regulating tumor glycolysis has become a popular research direction to reverse immune suppression. Most tumor cells obtain energy via aerobic glycolysis, which drives massive lactate buildup and acidifies the TME [9–13]. Inhibition of major enzymes involved in glycolysis such as lactate dehydrogenase A (LDHA) can reduce lactate production, which helps to alleviate microenvironmental acidosis, and restore the normal function of immune cells [14–16]. Diclofenac, a clinically approved anti-inflammatory drug, is known to suppress c-MYC expression, thereby inhibiting LDHA-mediated glycolysis and lactate transport [17]. Furthermore, diclofenac also exerts inhibitory effects on COX-2-related inflammatory cascades, which helps to alleviate the inflammatory response caused by therapeutic interventions [18–20]. Meanwhile, moderate restriction of pathological vascular overgrowth by nintedanib (NIB) achieves tumor vascular normalization and relieves intratumoral hypoxia, thereby boosting immune cell infiltration [21]. Importantly, vascular normalization is fundamentally different from conventional angiogenesis inhibition. Traditional anti-angiogenic strategies broadly ablate tumor vessels, aggravate hypoxia, and ultimately hinder immune infiltration. In comparison, NIB-mediated vascular remodeling selectively repairs disorganized, dysfunctional vessels while retaining normal vascular perfusion, which optimizes the tumor microenvironment to favor anti-tumor immune activation. Recent studies have confirmed that combining lactate clearance with vascular normalization can enhance the immune response and inhibit tumor progression. These findings further validate the therapeutic potential of these approaches in treatment [22]. The metal-coordination ability of Zn²⁺ offers a strategy for constructing multifunctional nanoplatforms that integrate the above therapeutic functions. Zn²⁺ can also target and block GLUT1 function, leading to effective inhibition of the glycolytic pathway [23, 24]. We hypothesize that integrating glycolysis inhibition, vascular normalization, and immune modulation within a single platform could achieve synergistic therapeutic effects. Specifically, glycolysis inhibition reduces lactate-mediated immunosuppression. Vascular normalization improves oxygenation and drug delivery. Crucially, the coordinating role of Zn²⁺ integrates these mechanisms, making them interdependent rather than operating in isolation: reduced lactate levels alleviate immunosuppression and improve immune cell responsiveness, which is further facilitated by enhanced vascular perfusion, enabling more efficient immune cell infiltration and drug delivery [25, 26]. Together, these processes act synergistically to establish a self-amplifying antitumor immune response.

Given the acidic intratumoral microenvironment of osteosarcoma, pH-responsive hydrogels serve as ideal local delivery carriers for solid tumors [27, 28]. Compared with conventional nanocarriers, they enable precise, tumor-targeted controlled drug release and offer significant advantages in the co-delivery of multiple functional therapeutics [29]. To realize this strategy, we developed a novel injectable pH-responsive hydrogel (OP@NIB-Zn-D780 NPs) for localized and sustained delivery of therapeutic agents. This Schiff-base crosslinked hydrogel can slowly release loaded NPs within the acidic TME, supporting efficient local NPs accumulation at tumor lesions. Upon laser irradiation, OP@NPs induces tumor cell damage while simultaneously disrupting metabolic and vascular homeostasis [30]. The coordinated regulation of these pathways effectively alleviates the ITME, enhances antitumor immunity, and suppresses lung metastasis.

**Scheme 1:**
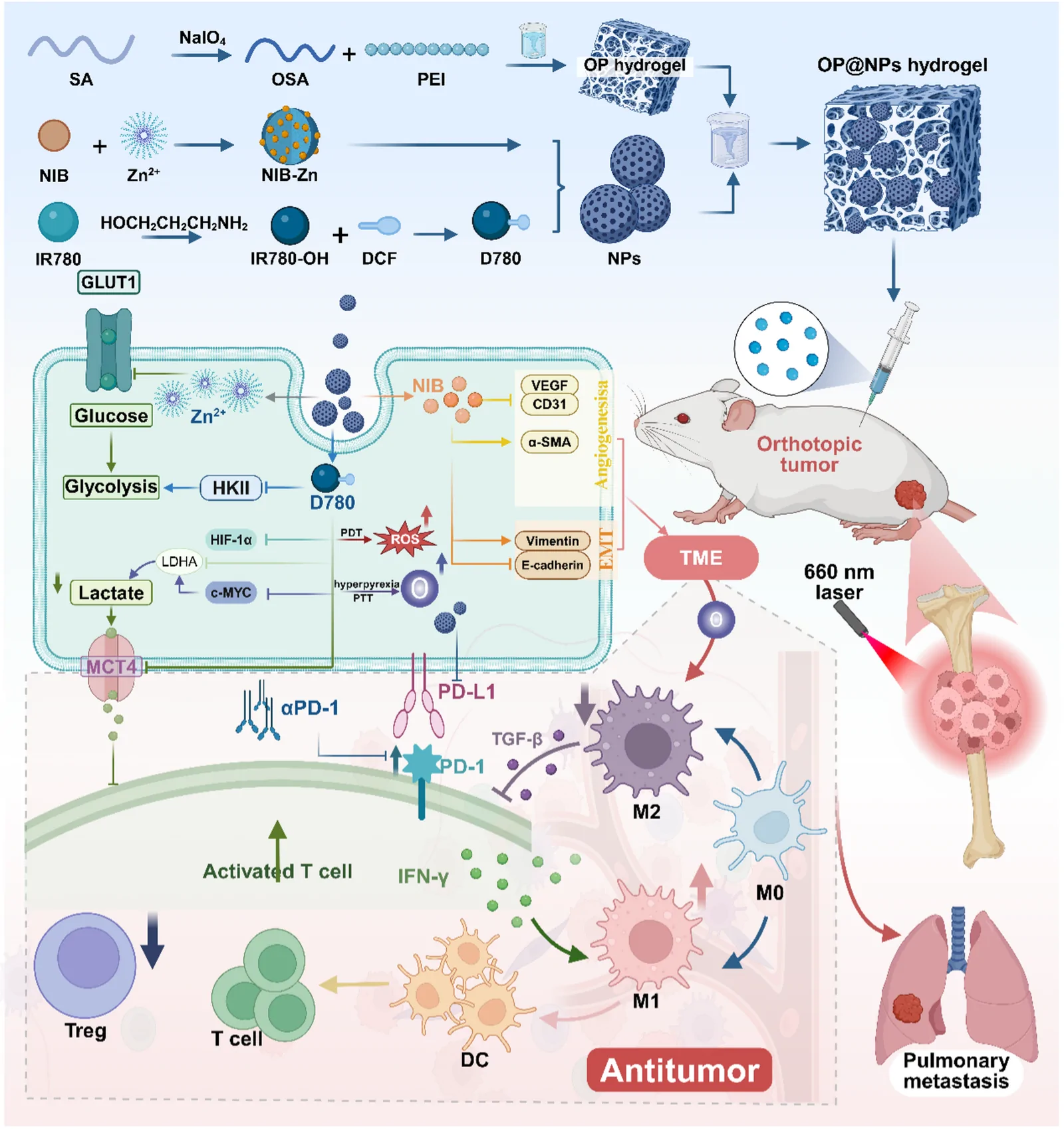
Schematic illustration of OP@NIB-Zn-D780 NPs influencing tumor metabolic mechanisms. Schematic illustration of OP@NIB-Zn-D780 NPs reshaping the ITME. In vivo therapeutic model of OP@NIB-Zn-D780 NPs

This work develops a hydrogel-based nanoplatform that combines phototherapy-triggered glycolysis inhibition and vascular normalization for osteosarcoma treatment. Benefiting from simple synthesis and biocompatible low-cost materials, the platform may support scaled preparation to some extent. Its capability for localized and controlled drug release lays a preliminary foundation for subsequent translational research. As an injectable formulation, this platform allows localized drug release, which may reduce invasiveness and systemic side effects. Collectively, the nanogel remodels the ITME, activates immune responses and inhibits pulmonary metastasis. This multi-target strategy may help relieve immunotherapy resistance and improve overall treatment efficacy (as illustrated in Scheme 1).

## Result

### Preparation and characterization of NIB-Zn-D780 NPs

**Fig. 1:**
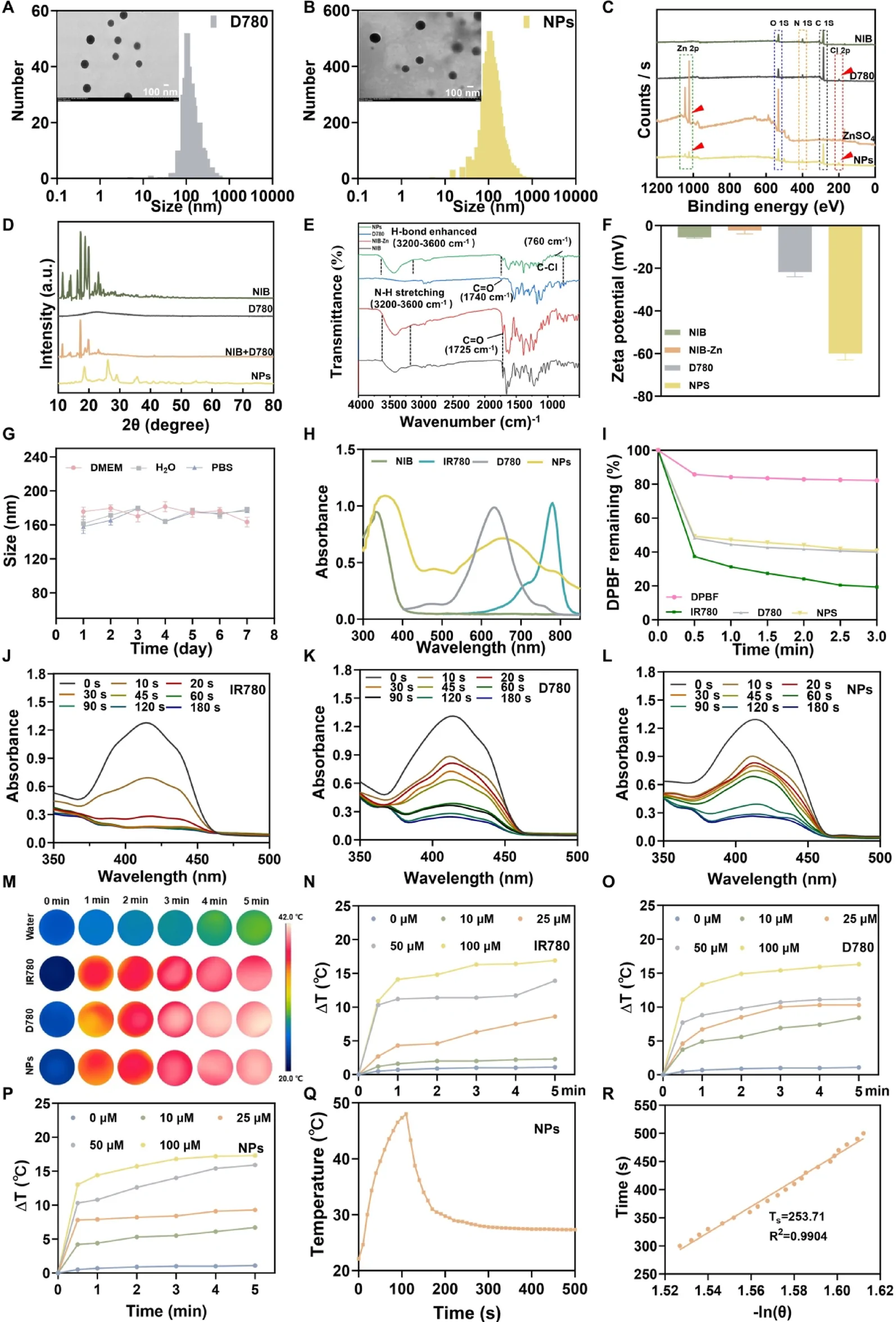
Preparation and characterization of NIB-Zn-D780 NPs. (**A-B**) TEM and size distribution of (**A**) D780 and (**B**) NPs. (**C**) XPS spectra of NIB, D780, ZnSO_4_, and NPs. (**D**) XRD patterns of NIB, D780, NIB + D780, and NPs. (**E**) FTIR spectra of NIB, NIB-Zn, D780, NPs, and OP@NPs. (**F**) Zeta potential of NIB, NIB-Zn, D780, and NPs. (**G**) Changes in the size of NPs over one week. (**H**) UV-vis absorption spectra of IR780, NIB, D780, and NPs. (**I**) Remaining levels of DPBF in various groups following laser irradiation. UV-vis absorption spectra for DPBF at various time intervals following laser irradiation for (**J**) IR780, (**K**) D780, and (**L**) NPs. (**M**) Thermal infrared images of IR780, D780, and NPs in water following laser exposure (1.0 W·cm⁻², 808 nm for IR780; 1.0 W·cm⁻², 660 nm for D780 and NPs). (**N-P**) Temperature changes of IR780, D780, and NPs at diverse dosages under laser irradiation (1.0 W·cm^− 2^, 660 nm, 5 min). (**Q**) Temperature rise and decrease profiles of NPs in water under laser irradiation for 110 s (λ = 660 nm, 1.0 W·cm⁻²). (**R**) Time linear profiles versus -ln(θ) measured in the cooling period of NPs. Each data point represents the mean ± SD of three independent experiments

IR780-OH was synthesized by esterifying IR780 with 3-Aminopropanol (Figure S1A), and the structural characteristics were verified using ^1^H NMR (Figure S1B). Next, sodium diclofenac was reacted with IR780-OH to produce D780, a compound with photothermal, photodynamic, and glycolysis-inhibitory functions (Figure S1C-D). NIB-Zn particles were prepared and combined with D780 to form spherical NPs through stirring. Hydrodynamic particle size analysis (NTA) showed that the diameters of NIB, NIB-Zn, D780, and NPs were 90, 100, 150, and 170 nm, respectively (Fig. 1A-B and Figure S2A-B). We characterized the morphology of D780 and NPs using transmission electron microscopy (TEM). The average diameters were approximately 140 and 180 nm (Fig. 1A-B), providing structural advantages for their application in nanomedicine delivery. UV-vis absorption confirmed the successful synthesis of NPs (Fig. 1H). The XPS spectra confirmed the successful synthesis of NIB-Zn-D780 NPs, as the sample simultaneously exhibited characteristic signals of NIB (O 1s/N 1s/C 1s), Zn (Zn 2p), and D780 (O 1s/C 1s/Cl 2p) (Fig. 1C). Compared with the physical mixture of NIB and D780, the XRD patterns of the NPs showed markedly attenuated characteristic peaks of NIB and the appearance of new diffraction signals, confirming the formation of an amorphous composite rather than a simple mixture (Fig. 1D). The FTIR spectrum of NPs showed a shifted C = O peak (from 1725 cm⁻¹ to 1740 cm⁻¹) and a broadened band at 3200–3600 cm⁻¹, indicating Zn²⁺ coordination with carbonyl groups and hydrogen bond formation. Moreover, the altered C-Cl signal further confirmed the involvement of D780 in the coordination network, demonstrating that the NPs were formed through coordination interactions rather than simple physical mixing (Fig. 1E). NPs’ stability was evaluated by measuring the zeta potential, which was approximately − 60 mV, indicating high stability (Fig. 1F). The NPs were then incubated in water, PBS, and serum-containing DMEM at 37 °C for 7 days (Fig. 1G). The results demonstrated minimal particle size variation, confirming the favorable colloidal stability of the nanoplatform under simulated in vivo conditions. Subsequently, photodynamic efficacy was assessed using a 1,3-diphenylisobenzofuran (DPBF) reagent (Fig. 1I-L and Figure S2C). After 3 min of laser irradiation, DPBF absorbance measurements showed that both free D780 and NPs produced obvious reactive oxygen species (ROS), which confirmed their favorable photodynamic capacity. Photothermal efficiency was assessed by measuring temperature changes during laser irradiation (Fig. 1M-P). NPs, IR780, and D780 exhibited concentration-dependent temperature elevation. NPs possessed photothermal conversion efficiency (Fig. 1Q-R), suggesting good performance for photothermal therapy (PTT).

### Preparation and characterization of nanocomposite hydrogel (OP@NPs)

**Fig. 2:**
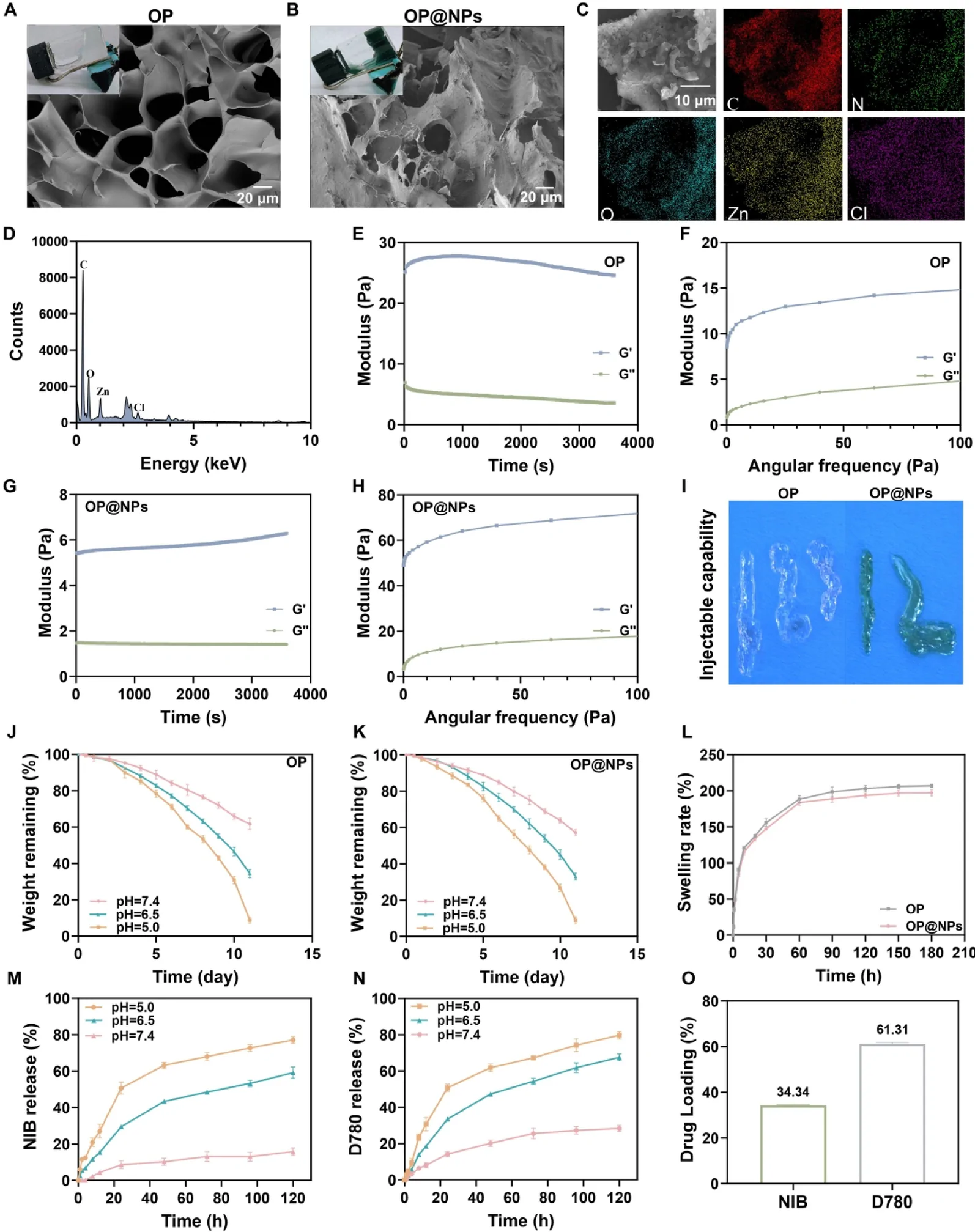
Preparation and characterization of OP@NPs. (**A-B**) Macroscopic images and SEM images of OP and OP@NPs hydrogel. (**C**) Elemental mapping and (**D**) EDS analysis of OP@NPs. (**E-H**) Rheological properties of OP and OP@NPs. (**I**) Macroscopic images of hydrogel injection. (**J-K**) Degradation profiles of OP and OP@NPs at different pH values. (**L**) Swelling profiles of hydrogels. (**M-N**) Release behavior of NIB and D780 at different pH values. (**O**) Drug loading efficiency of the OP@NPs system. Each data point represents the mean ± SD of three independent experiments

The pH-responsive hydrogel (OP) was synthesized by condensing aldehyde groups of oxidized sodium alginate (OSA) with amino groups of polyethyleneimine (PEI) under mild conditions. To enrich hydrogel functions and improve drug loading capacity, NPs were uniformly incorporated into the hydrogel network to prepare the OP@NPs composite hydrogel. Scanning electron microscopy (SEM) revealed a typical three-dimensional porous network, supporting uniform drug distribution (Fig. 2A-B). Energy-dispersive X-ray spectroscopy (EDS) and elemental mapping confirmed a uniform distribution of elements (C, N, O, Zn, Cl) within the OP@NPs hydrogel (Fig. 2C-D), consistent with NPs’ characteristics. These results verified the successful encapsulation and even distribution of NPs in the hydrogel, which benefited subsequent drug release and functional assays. Rheological testing showed a higher storage modulus (*G’*) than loss modulus (*G”*), indicating a stable structure suitable for long-term application (Fig. 2E-H). Syringe injection tests verified the excellent injectability of the prepared nanoplatform (Fig. 2I). Hydrogel degradation was investigated under physiological and acidic conditions (Fig. 2J-K). The hydrogel maintained high stability in PBS with negligible weight loss. By contrast, acidic surroundings greatly facilitated its degradation. This distinct pH-dependent property enableed targeted drug release in the acidic TME. Swelling tests showed that water absorption was rapid during the first 60 hours (Fig. 2L). This favorable swelling performance indicateed that it possessed good hydrophilicity and a porous network structure, which helped to promote molecular penetration and drug diffusion. In vitro release assays at pH 6.5 showed that NIB and D780 exhibited rapid release, with the cumulative drug release reaching 70% within 5 days (Fig. 2M-N). These results indicated that the hydrogel exhibited ideal controlled-release properties in an acidic environment. We further determined the loading efficiencies of NIB and D780 in the hydrogel, which provided foundational data for subsequent concentration quantification (Fig. 2O and Figure S2D-E). After peritumoral injection, the hydrogel retained most cargos locally at tumors while slowly releasing partial D780 and NIB into blood circulation. The two agents showed synchronized plasma profiles, peaking at day 4 and remaining detectable for 14 days (Figure S2F-G). Overall, the pH-responsive behavior and excellent physicochemical characteristics of OP@NPs made it a promising platform for biomedical drug delivery.

### In vitro cytotoxicity and anti-metastatic mechanisms of OP@NPs

**Fig. 3:**
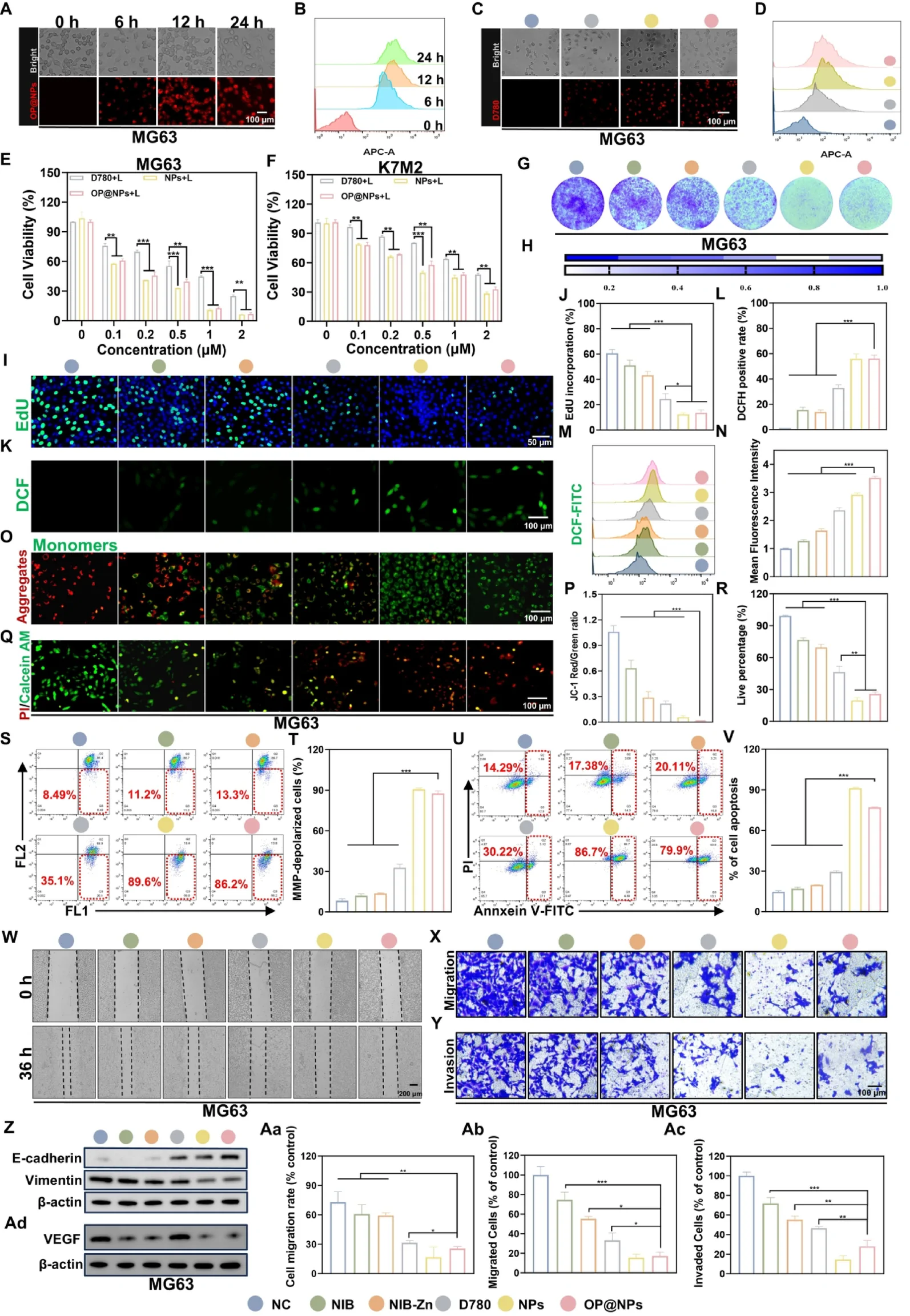
In vitro cytotoxicity and anti-metastatic mechanisms of OP@NPs. Cellular uptake of OP@NPs in MG63 cells assessed via (**A**) fluorescent micrographs and (**B**) flow cytometric analysis. (**C**) Cellular uptake of D780, NPs, and OP@NPs in MG63 cells. (**D**) Flow cytometric analysis for the cellular uptake of D780, NPs, and OP@NPs in MG63 cells. Cell viability of (**E**) MG63 and (**F**) K7M2 cells after treatment with D780, NPs, and OP@NPs under laser irradiation (*n* = 3, 10 W·cm⁻², 660 nm, 30 s, Two-way ANOVA). (**G**) Representative photographs and (**H**) quantitative analysis of colony formation in MG63 cells under different conditions (*n* = 3, Student’s *t*-test). (**I**) Fluorescent images and (**J**) quantitative analysis of EdU labeling assay in MG63 cells under different conditions (*n* = 3, Student’s *t*-test). (**K**) Representative fluorescent images and (**L**) statistical analysis of DCFH-DA staining in MG63 cells under different conditions (*n* = 3, Student’s *t*-test). (**M-N**) Flow cytometric analysis of DCFH-DA in MG63 cells under different conditions (*n* = 3, Student’s *t*-test). (**O**) Fluorescent images and (**P**) analysis of JC-1 staining in MG63 cells under different conditions (*n* = 3, Student’s *t*-test). (**Q**) Live/dead staining and (**R**) analysis of MG63 cells under different conditions (*n* = 3, Student’s *t*-test). (**S-T**) JC-1 flow cytometric analysis and (**U-V**) apoptosis flow cytometric analysis of MG63 cells under different conditions (*n* = 3, Student’s *t*-test). (**W**) Wound healing assay for migration evaluation of MG63 cells. (**X**) Migration and (**Y**) invasion abilities of MG63 cells measured via Transwell assays. Quantitative analysis of (**Aa**) wound healing, (**Ab**) migration, and (**Ac**) invasion (*n* = 3, Student’s *t*-test). Detection of (**Z**) EMT-related proteins and (Ad) VEGF expression in MG63 cells (*n* = 3). Each data point represents the mean ± SD of three independent experiments. (* *P* < 0.05; ** *P* < 0.01; *** *P* < 0.001)

Efficient drug accumulation in tumor cells is critical for achieving therapeutic efficacy [31–33]. To evaluate the cellular uptake and toxicity of OP@NPs, we examined their internalization in MG63 and K7M2 cells using flow cytometry and fluorescence imaging. Over a 0–24 h period, both methods showed time-dependent increases in internalization (Fig. 3A-B and Figure S3A-B). Notably, obvious aggregation of NPs was observed after 12 h, indicating their high efficiency in cellular uptake. Compared with the control and D780 groups, the OP@NPs group showed brighter fluorescence signals, confirming efficient drug delivery (Fig. 3C-D and Figure S3C-D). To further investigate the cytotoxicity of OP@NPs, MTT assays were used to evaluate the effects of different treatments (Fig. 3E-F and Figure S3E-F). Under laser irradiation (808 nm for IR780, 660 nm for D780/NPs/OP@NPs), we observed a lower *IC₅₀* for OP@NPs. Under non-irradiated conditions, the *IC₅₀* values for D780, NPs, and OP@NPs were all relatively high. These results indicated that OP@NPs exhibited potent cytotoxic effects under laser irradiation. Functional assays, including EdU labeling and clonogenic assays, revealed inhibition of cell proliferation in MG63 and K7M2 cells (Fig. 3G-J and Figure S3G-J). ROS assays confirmed that OP@NPs increased intracellular ROS levels to induce oxidative stress, thereby enhancing anti-tumor activity (Fig. 3K-N and Figure S3K-N). JC-1 mitochondrial potential assays indicated depolarization of the mitochondrial membrane, suggesting mitochondrial dysfunction and triggering apoptosis (Fig. 3O-P, S-T and Figure S3O-P, S-T). Consistently, live/dead staining and apoptosis flow cytometry analysis further confirmed increased apoptotic and necrotic cell populations (Fig. 3Q-R, U-V and Figure S3Q-R, U-V). Regarding metastasis, functional assays (wound healing, migration, and invasion) validated OP@NPs’ inhibition of tumor cell migration and invasion (Fig. 3W-Y, Aa-Ac and Figure S3W-Y, Aa-Ac). Western blotting revealed that OP@NPs upregulated E-cadherin and downregulated Vimentin and VEGF (Fig. 3Z, Ad and Figure S3Z, Ad). This suggested that OP@NPs reduced metastasis by inhibiting EMT-associated phenotypes [34, 35]. In summary, OP@NPs effectively eliminated tumor cells and inhibited their metastatic potential via a combination of chemotherapy, photodynamic therapy, and photothermal therapy.

### Metabolic reprogramming of glycolysis by OP@NPs

**Fig. 4:**
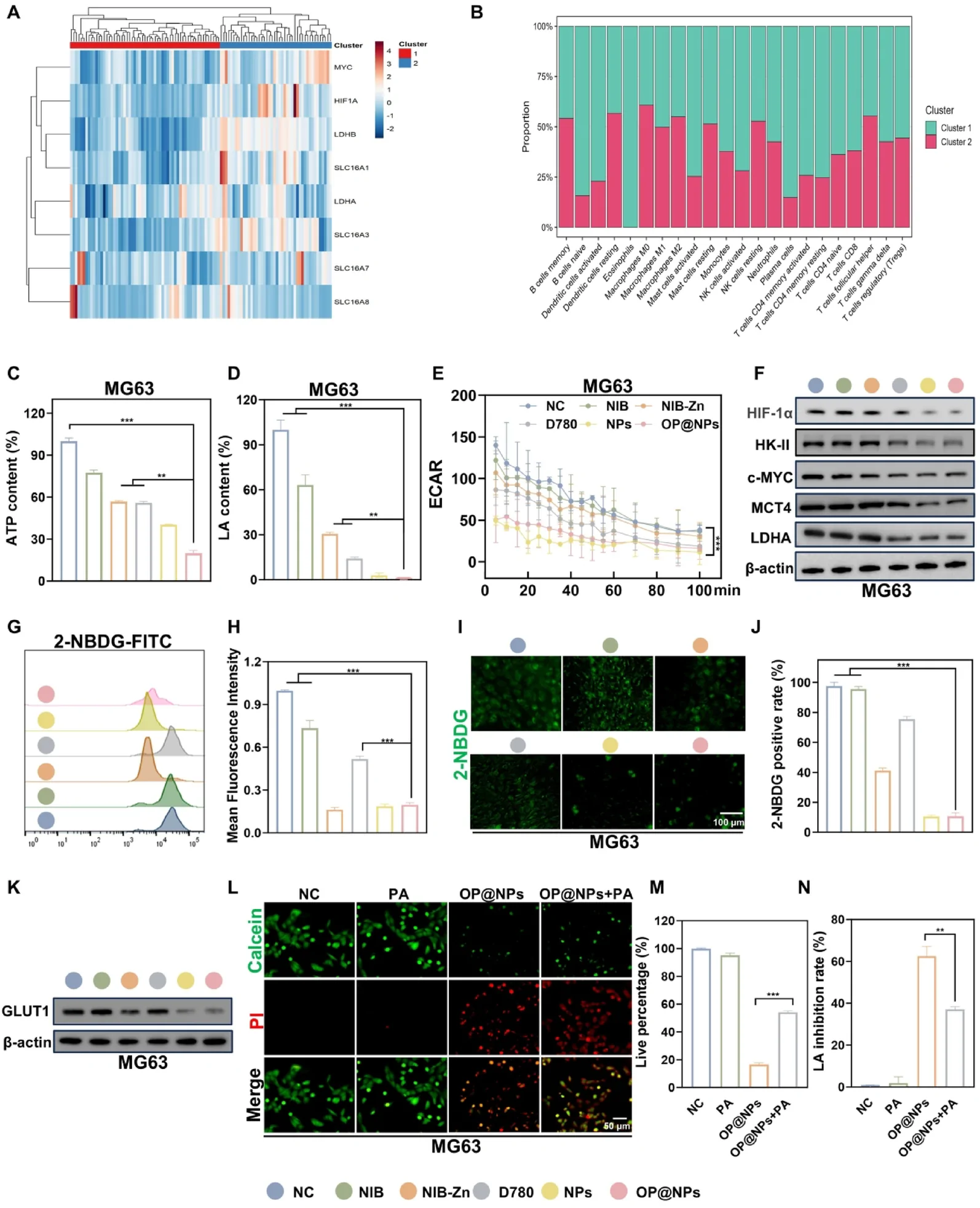
Metabolic reprogramming of glycolysis by OP@NPs. The low lactate metabolism group is represented by Cluster 1, while the high lactic acid metabolism group is represented by Cluster 2. (**A**) Heatmap of glycolysis-related gene expression. (**B**) The ratio of immune cells in the two clusters. (**C**) ATP, (**D**) LA, and (**E**) ECAR assays of MG63 cells after treatment with NIB, NIB-Zn, D780, NPs, and OP@NPs under laser irradiation (*n* = 3, 1.0 W·cm⁻², 660 nm, 30 s, Student’s *t*-test). (**F**) Immunoblot analysis of lactate metabolism-related proteins in MG63 cells (*n* = 3). (**G-H**) Flow cytometric analysis, (**I**) representative fluorescence micrographs, and (**J**) quantitative analysis of 2-NBDG staining in MG63 cells (*n* = 3, Student’s *t*-test). (**K**) Immunoblot analysis of GLUT1 expression (*n* = 3). (**L**) Live/dead staining of MG63 cells and (**M**) analysis with or without pyruvate supplementation (*n* = 3, Student’s *t*-test). (**N**) LA inhibition rate in MG63 cells with or without pyruvate supplementation (*n* = 3, Student’s *t*-test). Each data point represents the mean ± SD of three independent experiments. (* *P* < 0.05; ** *P* < 0.01; *** *P* < 0.001)

Besides functioning as a metabolic substrate, lactate also serves as a signaling molecule regulating both adaptive and innate immune responses [36]. To explore the immunological relevance of lactate metabolism, we first performed bioinformatics analysis. Results from these analyses revealed that elevated lactate levels correlated with immunosuppressive states (Fig. 4A-B and Figure S4A) [9, 37, 38]. Indeed, tumor metabolism drives growth and metastasis, particularly in hypoxic and nutrient-deprived TMEs, where dysregulated glycolysis and lactate metabolism facilitate immune evasion and therapeutic resistance, thereby forming a vicious cycle that accelerates tumor progression [39–42]. Further metabolic analysis showed that OP@NPs synergistically suppressed ATP production (Fig. 4C and Figure S4B), lactate generation (Fig. 4D and Figure S4C), and extracellular acidification rates (ECAR) (Fig. 4E and Figure S4D). These results demonstrated that OP@NPs inhibited tumor glycolysis, relieved intratumoral acidosis, and realized tumor metabolic reprogramming. Key lactate metabolism proteins, including LDHA, MCT4, c-MYC, HK-II, and HIF-1α, were downregulated after OP@NPs treatment, confirming their modulation of lactate metabolism (Fig. 4F and Figure S4E). 2-NBDG uptake assays showed that OP@NPs reduced glucose uptake, which compounded glycolysis inhibition and synergistically impacted cellular energy metabolism (Fig. 4G-J and Figure S4F-I). Western blots confirmed that OP@NPs suppressed GLUT1 expression (Fig. 4K and Figure S4J).

To verify that OP@NPs actively regulate lactate metabolism, we replenished pyruvate (PA) to restore the lactate metabolic pathway and examined cell death and lactate production [43]. As shown in Fig. 4L-N and Figure S4K-M, PA alone had no significant effect on cell viability. OP@NPs treatment alone increased red fluorescence (dead cells) and reduced lactate generation. In contrast, co-incubation with OP@NPs and PA partially reversed these effects, rescuing cell viability and attenuating the lactate inhibition rate. These results indicated that exogenous PA could relieve the lactate metabolic blockade and alleviate OP@NPs-induced cytotoxicity, suggesting that OP@NPs exerted a direct metabolic intervention on lactate metabolism that was at least partially independent of cell death.

### Vascular remodeling and immune activation by OP@NPs

**Fig. 5:**
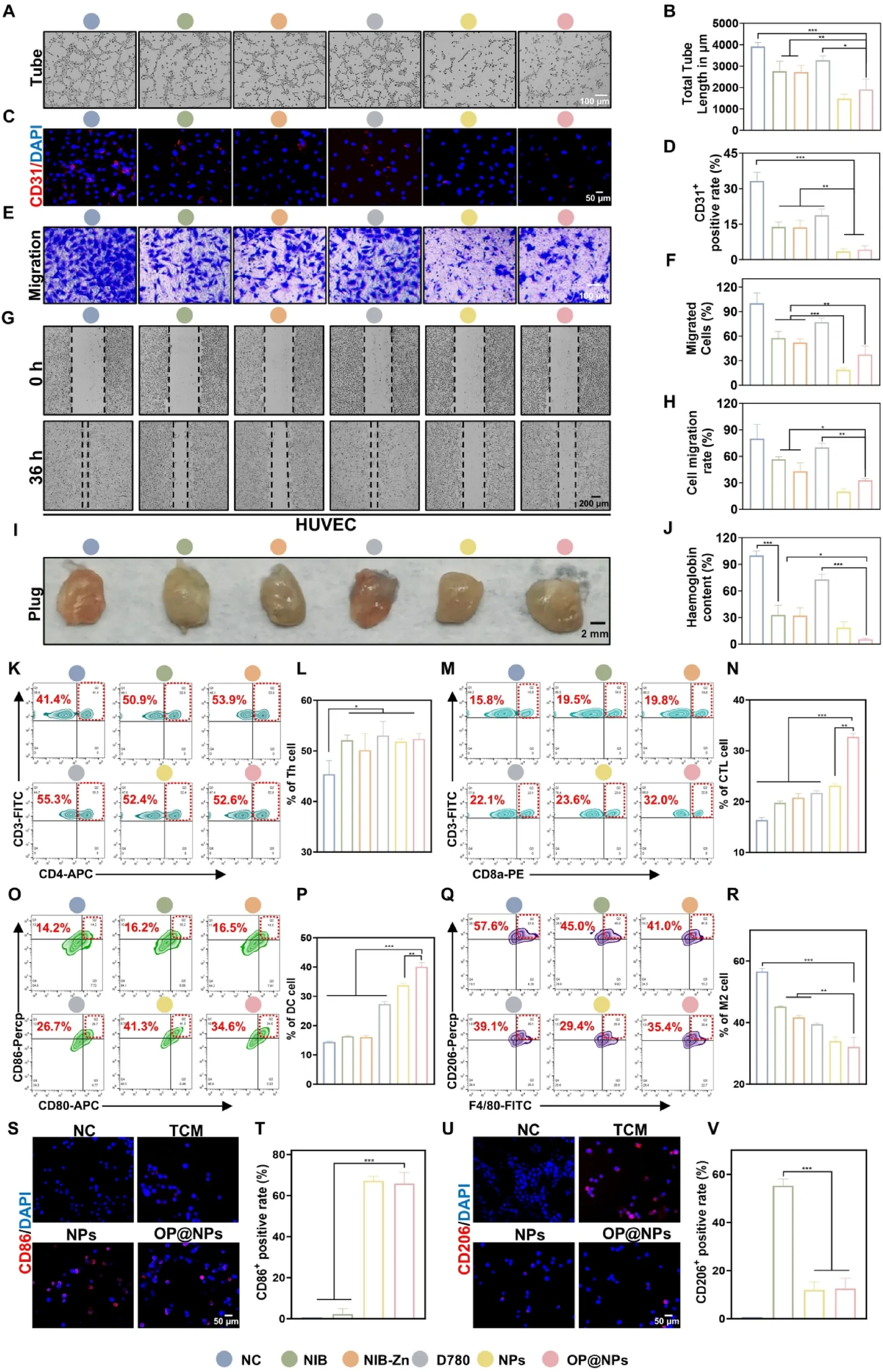
Vascular remodeling and immune activation by OP@NPs. (**A-B**) Representative images of tube formation in HUVECs after treatment with NIB, NIB-Zn, D780, NPs, and OP@NPs under laser irradiation (*n* = 3, 1.0 W·cm⁻², 660 nm, 30 s, Student’s *t*-test). (**C-D**) Immunofluorescence images and analysis of HUVECs stained for CD31 (*n* = 3, Student’s *t*-test). (**E-F**) Migration evaluation of HUVECs by Transwell assays (*n* = 3, Student’s *t*-test). (**G-H**) Migration evaluation of HUVECs by wound healing assays (*n* = 3, Student’s *t*-test). (**I-J**) Macroscopic images and analysis of Matrigel plug assays (*n* = 3, Student’s *t*-test). Flow cytometric detection of (**K-N**) T cells (CD3^+^CD4^+^/CD3^+^CD8^+^), (**O-P**) DCs (CD11c^+^CD80^+^CD86^+^), and (**Q-R**) M2 TAMs (CD11b^+^CD206^+^F4/80^+^) in the spleen (*n* = 3, Student’s *t*-test). Immunofluorescence images and analysis of (**S-T**) M1 (CD86^+^) and (**U-V**) M2 (CD206^+^) macrophage markers (*n* = 3, Student’s *t*-test). Each data point represents the mean ± SD of three independent experiments. (* *P* < 0.05; ** *P* < 0.01; *** *P* < 0.001)

Abnormal vascular proliferation not only impairs nutrient delivery but also restricts immune cell infiltration, with dysfunctional tumor vascular endothelial cells impeding this process—especially the penetration of cytotoxic T cells into tumor tissues [44]. It should be emphasized that this beneficial effect relies on vascular normalization rather than non-selective elimination of neovessels. Conventional blind angiogenesis inhibition tends to worsen hypoxia and block immune trafficking, while moderate regulation of pathological vessels repairs vascular function to relieve hypoxia and facilitate immune infiltration, thus augmenting antitumor immunotherapeutic efficacy. Subsequently, in vitro angiogenesis-related assays were carried out. Tube formation assays revealed that OP@NPs inhibited abnormal vascular formation (Fig. 5A-B). Immunofluorescence staining of the endothelial marker CD31 was performed. A reduction in CD31-positive vascular structures was observed following OP@NPs treatment (Fig. 5C-D). Wound healing and migration assays also verified the inhibitory effect of OP@NPs on endothelial cell migration (Fig. 5E-H). Moreover, in vivo plug assays showed that OP@NPs inhibited excessive angiogenesis (Fig. 5I-J). Consistently, Western blot analysis in previous sections demonstrated downregulation of HIF-1α and VEGF expression, supporting alleviation of hypoxia and suppression of pro-angiogenic signaling. Given the potential of lactate metabolism inhibition and anti-angiogenic effects to reactivate antitumor immunity, we further examined the immunomodulatory activity of OP@NPs via in vitro immune co-culture assays (Figure S5A). ELISA analysis quantitatively demonstrated elevated levels of IL-2, TNF-α, IFN-γ, GZMB and PRF1, accompanied by reduced TGF-β expression (Figure S5B). Furthermore, we examined immune function markers in splenic and bone marrow immune cells, including Th cells, CTL cells, Treg cells, dendritic cells (DCs), NK cells, and M1/M2 macrophages (Fig. 5K-R and Figure S5C-N). OP@NPs elevated the proportions of CD4⁺ and CD8⁺ T cells, which served essential functions in eliminating tumor cells [45, 46]. OP@NPs promoted polarization of macrophages toward the M1 phenotype. M1 macrophages improved anti-tumor immune responses through the release of proinflammatory cytokines, including IL-2, TNF-α, and IFN-γ [47, 48]. To further validate macrophage polarization at the cellular level, RAW264.7 cells were cultured in conditioned medium derived from osteosarcoma cells to induce polarization, followed by treatment with OP@NPs. Immunofluorescence staining showed upregulated CD86 (M1 marker) and downregulated CD206 (M2 marker) in the OP@NPs-treated group (Fig. 5S-V), which confirmed macrophage polarization and contributed to immune reprogramming in the TME. OP@NPs also enhanced dendritic cell activation, which further supported improved antigen presentation and T cell priming [49, 50]. Collectively, these results indicated that OP@NPs induced vascular remodeling and immune activation within the TME. Notably, the improved vascular architecture may facilitate immune cell infiltration and functional activation, suggesting a mechanistic linkage between these two processes [51]. 

### Antitumor effects of OP@NPs in orthotopic osteosarcoma

**Fig. 6:**
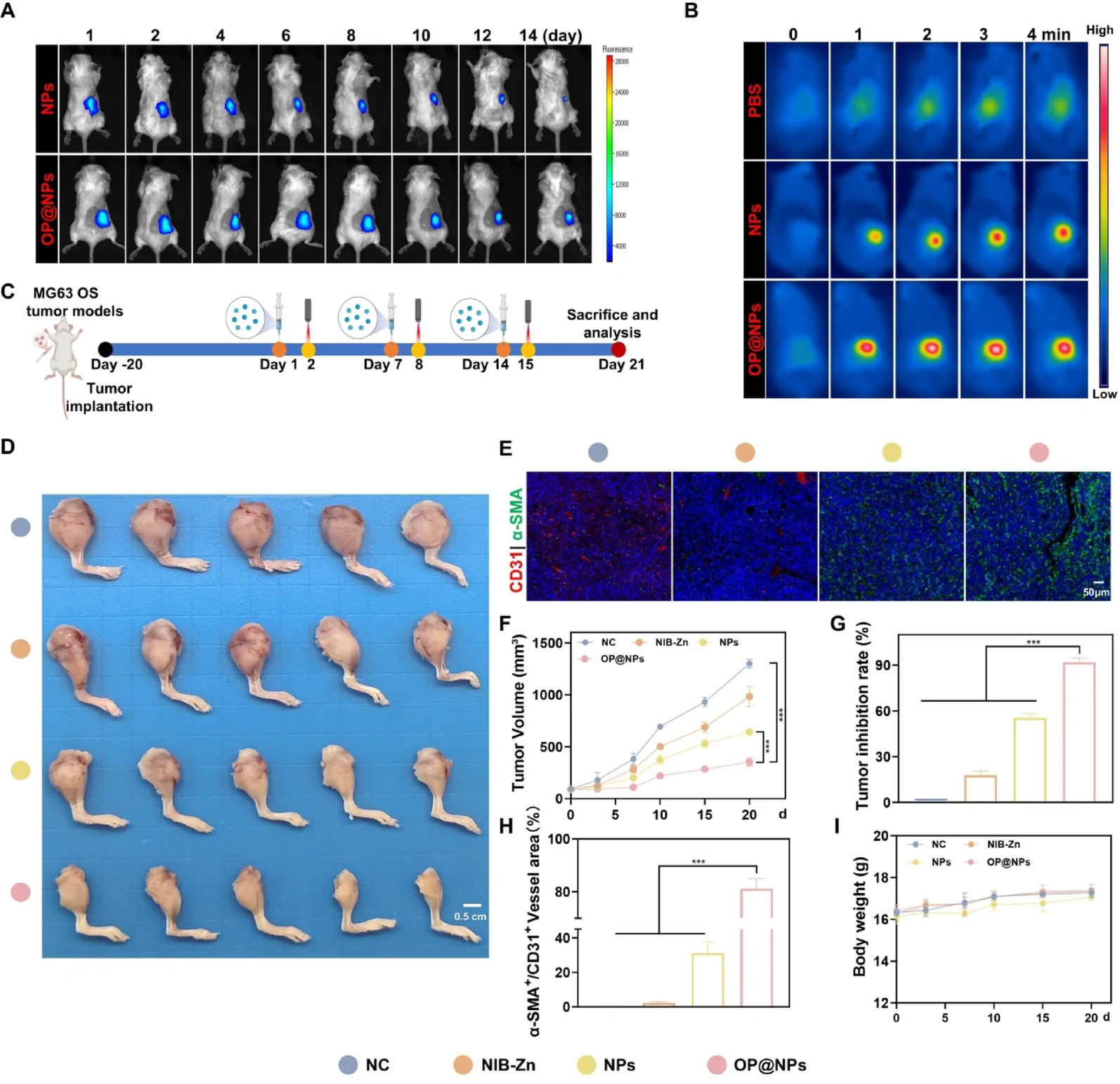
Antitumor effects of OP@NPs in orthotopic osteosarcoma. (**A**) Fluorescence analysis of drug metabolism in subcutaneously injected mice. (**B**) Thermal imaging of mice in different groups (660 nm, 1.0 W·cm^− 2^, 30 s). (**C**) Schematic illustration of the orthotopic tumor therapy technique in vivo. (**D**) Images of orthotopic tumors from different groups (*n* = 5). (**E**) Immunofluorescence staining of CD31 and α-SMA in tumor tissues (*n* = 3). (**F**) Tumor volume of each group at different time points (*n* = 5, Student’s *t*-test). (**G**) Tumor growth inhibition rate in each group (*n* = 5, Student’s *t*-test). (**H**) Quantitative analysis of α-SMA⁺/CD31⁺ ratio in tumor tissues (*n* = 3, Student’s t-test). (**I**) Dynamic changes in body weight of mice from different treatment groups (*n* = 5). Each data point represents the mean ± SD. (*** *P* < 0.001)

To investigate the in vivo retention and distribution of OP@NPs, intravital imaging was applied to monitor the subcutaneous distribution. NPs exhibited peak fluorescence on day 1 post-injection, whereas OP@NPs reached maximum intensity on day 6 and remained detectable for over 10 days (Fig. 6A), indicating prolonged retention. Based on this profile, laser irradiation (660 nm, 1 W·cm⁻², 4 min) was applied to the OP@NPs group on day 6. The temperature at the injection site increased to approximately 60.0 °C (Fig. 6B), confirming efficient photothermal conversion and suitability for photothermal therapy. To assess the overall therapeutic efficacy, we established an orthotopic osteosarcoma mouse model (Fig. 6C). This model faithfully reconstructed the primary TME, including hypoxia, abnormal vascular architecture, and typical local invasive phenotypes of human osteosarcoma [52]. Subsequently, we tracked tumor progression over a 21-day period (Fig. 6F). All treatment groups showed tumor-suppressing effects, with the OP@NPs group showing the most significant results (Fig. 6G). Gross images of tibial orthotopic tumors further verified the favorable therapeutic outcomes of OP@NPs (Fig. 6D). This improvement in therapeutic efficacy may be attributed to the combined photothermal/photodynamic effects and vascular modulation. Subsequently, we focused on tumor angiogenesis, which played a critical role in osteosarcoma progression. Abnormal angiogenesis and increased vascular permeability in the TME frequently accelerated tumor proliferation and metastasis [53, 54]. Dual immunofluorescent labeling was utilized to identify markers of vascular normalization in tumor tissues (Fig. 6E). The results showed a decrease in CD31 (vascular endothelial marker) and an increase in α-SMA (vascular smooth muscle marker) in the OP@NPs-treated group, indicating improved vascular structure. Quantitative analysis further demonstrated a significantly increased α-SMA/CD31 ratio in the OP@NPs group (Fig. 6H), supporting their role in promoting tumor vascular normalization [55, 56]. Biosafety was assessed by monitoring body weight during treatment (Fig. 6I). Overall, OP@NPs effectively suppressed tumor growth while promoting vascular normalization and maintaining favorable biosafety. Vascular normalization may enhance immune cell infiltration and metabolic regulation within the TME, thereby providing a mechanistic basis for subsequent systemic immune modulation.

### Anti-tumor immunity activation and biosafety of OP@NPs in vivo

**Fig. 7:**
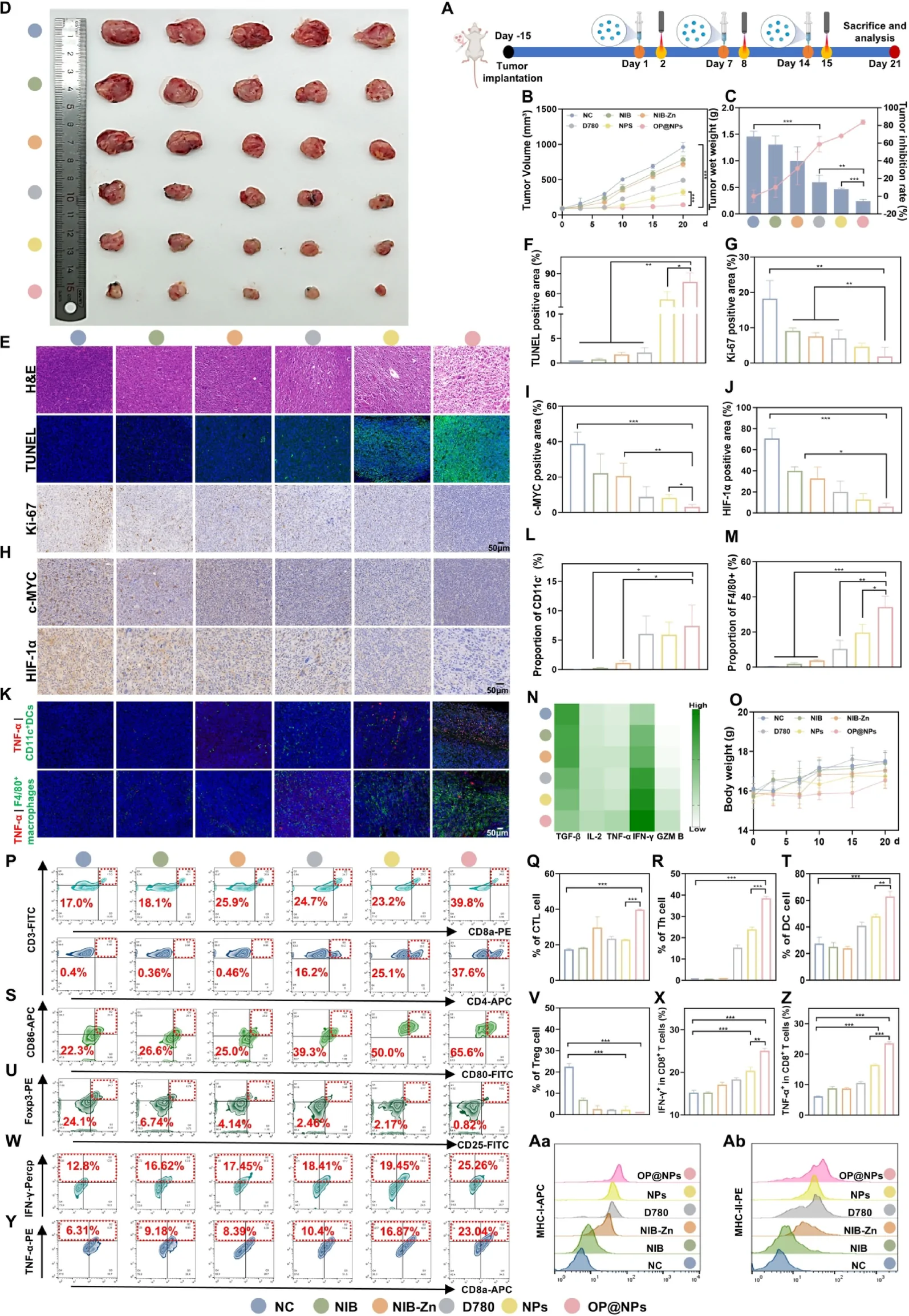
Anti-tumor immunity activation and biosafety of OP@NPs in vivo. (**A**) Schematic illustration of in vivo tumor treatment strategies. (**B**) Tumor volumes of each group at different time points (*n* = 5, Student’s *t*-test). (**C**) Tumor weight and growth inhibition rates in different groups (*n* = 5, Student’s *t*-test). (**D**) Representative images of tumors in different groups (*n* = 5). (**E-G**) Evaluation of tumor-suppressive effects: H&E staining, TUNEL staining, and Ki-67 staining (*n* = 3, Student’s *t*-test). (**H-J**) Immunohistochemical staining of lactate metabolism-related markers: c-MYC and HIF-1α (*n* = 3, Student’s *t*-test). (**K-M**) Dual-immunofluorescence staining of immune activation-related markers: TNF-α⁺, CD11c⁺ DCs and TNF-α⁺, F4/80^+^ macrophages (*n* = 3, Student’s *t*-test). (**N**) TGF-β, IL-2, TNF-α, IFN-γ, and GZM B expression in the serum detected by ELISA (*n* = 3). (**O**) Dynamic changes in mouse body weight (*n* = 5). Flow cytometry results of (**P-R**) T cells (CD3^+^CD4^+^/CD3^+^CD8^+^), (S-T) DCs (CD11c^+^CD80^+^CD86^+^), (**U-V**) Tregs (CD4^+^CD25^+^Foxp3^+^), (**W-X**) IFN-γ^+^ in CD8⁺ T cells, (**Y-Z**) TNF-α^+^ in CD8⁺ T cells and (**Aa**-**Ab**) MHC-I and MHC-II expression on CD11c⁺ DCs following indicated treatments in tumor (*n* = 3, Student’s *t*-test). Each data point represents the mean ± SD. (* *P* < 0.05; ** *P* < 0.01; *** *P* < 0.001)

To further evaluate systemic anti-tumor immunity independent of the orthotopic TME, a subcutaneous osteosarcoma model was established (Fig. 7A). Tumor-growth monitoring revealed significant inhibition in all treated groups, with the most pronounced effect observed in the OP@NPs group (Fig. 7B-C). Macroscopic tumor images further confirmed reduced tumor burden (Fig. 7D). Histological analysis showed extensive necrosis in OP@NPs-treated tumors (Fig. 7E), accompanied by increased apoptosis and reduced proliferation, as evidenced by TUNEL and Ki-67 staining (Fig. 7F-G). At the metabolic level, OP@NPs suppressed glycolysis-associated signaling pathways. Immunohistochemical analysis showed reduced expression of c-MYC and HIF-1α in tumor tissues (Fig. 7H-J). Consistently, OP@NPs modulated immune responses through metabolic reprogramming of the TME. Immunofluorescence staining showed increased infiltration of CD11c⁺ dendritic cells and F4/80⁺ macrophages, along with elevated TNF-α expression in tumor tissues (Fig. 7K-M and Figure S6A-B). Serum cytokine levels were assessed by ELISA, showing increased levels of IL-2, TNF-α, IFN-γ, and GZMB, while TGF-β was significantly reduced (Fig. 7N), indicating a shift towards a pro-inflammatory immune state. Flow cytometric analysis of tumor and spleen tissues revealed significant immune remodeling (Fig. 7P-V and Figure S6C-Q), including increased CD4⁺ T cells, CD8⁺ T cells, dendritic cells and M1 macrophages, along with decreased Treg and M2 populations. Notably, the secretion levels of IFN-γ and TNF-α by CD8⁺ T cells were markedly elevated, which verified the functional activation and tumor-killing capacity of cytotoxic T cells (Fig. 7W-Z). Meanwhile, the upregulated surface expression of MHC-I and MHC-II on dendritic cells further demonstrated the enhanced antigen-presenting function of DCs (Fig. 7Aa-Ab). Overall, OP@NPs modulated the ITME by suppressing glycolysis-associated metabolism and enhancing innate and adaptive immune responses, thereby promoting anti-tumor immunity in vivo. Together with the vascular normalization observed in the orthotopic model (Fig. 6), these findings indicated that vascular normalization and metabolic reprogramming synergistically promoted a pro-inflammatory TME, thereby suppressing metastatic dissemination.

Body weight was tracked during treatment with no obvious changes (Fig. 7O). Histological examination of organs (heart, liver, spleen, lung, and kidney) showed normal morphology (Figure S6R). Hemolysis assays indicated negligible erythrocyte damage (Figure S6S), and blood biochemical parameters (AST, CRE, URE) remained within normal ranges (Figure S6T-V). Collectively, OP@NPs exerted favorable immunotherapeutic effects and good biosafety, which provided a basis for further translational research.

### Vascular normalization drives antitumor immune activation in vivo

**Fig. 8:**
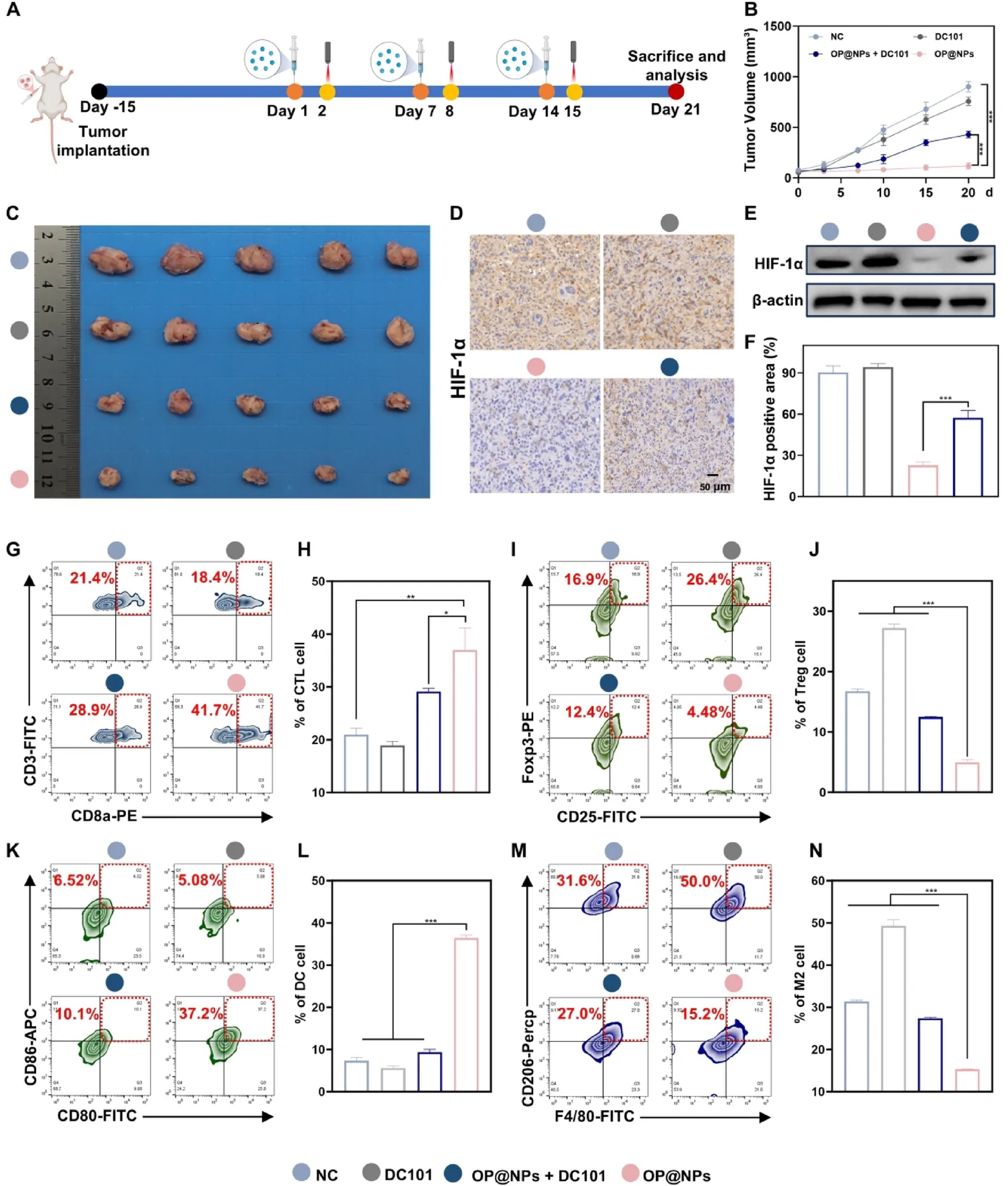
Vascular normalization drives antitumor immune activation in vivo. (**A**) Schematic illustration of the subcutaneous osteosarcoma model timeline with DC101 intervention. (**B**) Tumor growth curves for mice across treatment groups (*n* = 5, Student’s *t*-test). (**C**) Macroscopic images of tumors (*n* = 5). (**D**) Immunohistochemical staining of HIF-1α in tumor tissues. (**E**) Western blot analysis of HIF-1α expression in tumor tissues (*n* = 3). (**F**) Quantitative analysis of HIF-1α immunohistochemical staining in tumor tissues (*n* = 3, Student’s *t*-test). Flow cytometric analysis and quantification of (**G-H**) CD3⁺CD8⁺ CTLs, (I-J) CD25⁺Foxp3⁺ Tregs, (**K-L**) CD80⁺CD86⁺ DCs, and (**M-N**) CD206⁺ M2 macrophages in tumor. Each data point represents the mean ± SD. (** *P* < 0.01; *** *P* < 0.001)

To determine whether vascular remodeling acted as an upstream event regulating metabolic reprogramming and antitumor immunity, a rescue assay was conducted with DC101, a specific VEGFR-2 blocking antibody, in a subcutaneous osteosarcoma model (Fig. 8A). Tumor growth curves (Fig. 8B), representative photographs of tumors (Fig. 8C) and tumor inhibition rates (Figure S7A) showed that OP@NPs suppressed tumor progression relative to control and DC101 groups. Notably, the combined use of DC101 and OP@NPs partially attenuated the antitumor effect of OP@NPs. This indicated that OP@NPs depended on functional vascular normalization rather than non-specific vascular inhibition to exert their therapeutic efficacy. Immunohistochemical staining and Western blot analysis further revealed that OP@NPs treatment reduced the expression of HIF-1α (Fig. 8D-F). This reduction was reversed in the OP@NPs + DC101 group, demonstrating that DC101 blocked the hypoxia-relieving effect of OP@NPs. Thus, vascular normalization acted upstream to alleviate intratumoral hypoxia. Flow cytometry detection of tumor-infiltrating immune cells showed that OP@NPs increased the proportions of CD4⁺ T cells, CD8⁺ T cells, dendritic cells, and M1 macrophages, while reducing the proportion of Treg and M2 populations (Fig. 8G-N and Figure S7C-F). These immune modulatory alterations were diminished or reversed following combined treatment with OP@NPs and DC101. These rescue experiments showed that OP@NPs boosted immune activity by normalizing blood vessels, not by simply blocking them. Body weight monitoring confirmed that no obvious toxicity was observed in any group (Figure S7B).

Given the above evidence that OP@NPs remodelled tumor vasculature and reprogram cellular metabolism to reshape the TME, we further explored whether such favorable changes could improve the responsiveness of osteosarcoma to PD-1 blockade immunotherapy. We speculated that OP@NPs might confer higher sensitivity of osteosarcoma to PD-1 inhibitors via the aforementioned metabolic reprogramming and vascular remodeling. In vivo combination experiments confirmed that OP@NPs strengthened the anti-tumor activity of PD-1 inhibitor (Figure S8A-D). Elevated PD-1 and PD-L1 expression was observed in tumors after co-treatment (Figure S8E-K), along with potent immune activation and T cell expansion in tumors and spleens (Figure S8L-Q). Overall, OP@NPs remodelled vasculature and metabolism to improve tumor immunogenicity and modulate immune checkpoints, which greatly increased the responsiveness of osteosarcoma to PD-1 blockade therapy.

### OP@NPs effectively inhibit osteosarcoma lung metastasis

**Fig. 9:**
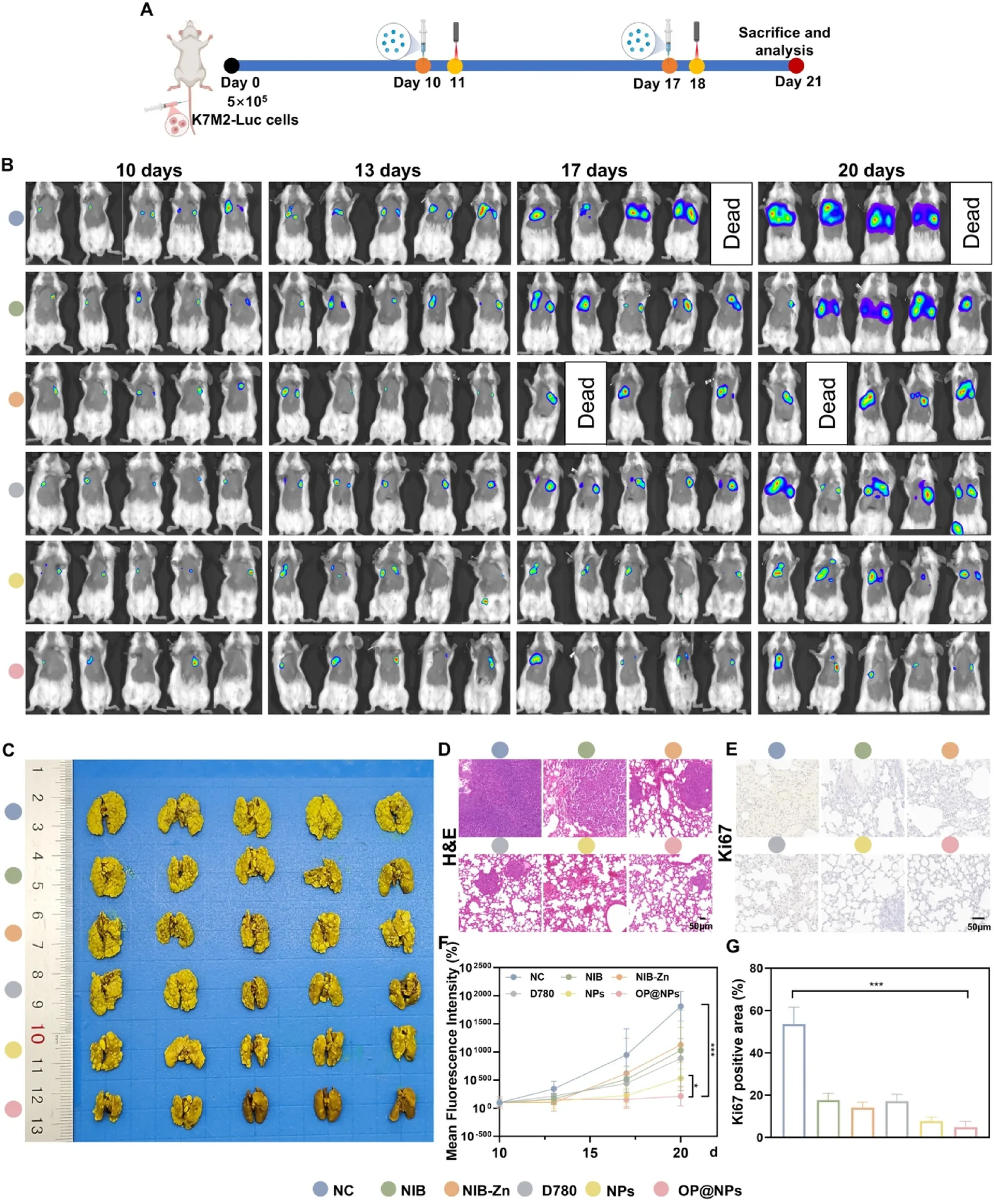
OP@NPs inhibit osteosarcoma pulmonary metastasis. (**A**) Schematic diagram of the tail vein injection mouse model for establishing lung metastasis. (**B**) Bioluminescence images of lung metastases at different time points in mice (*n* = 5). (**C**) Representative images of metastatic nodules in isolated lung tissues from mice (*n* = 5). (**D**) H&E staining of mouse lung metastatic nodules (*n* = 3). (**E**) Immunohistochemical analysis of Ki-67 expression in lung metastatic nodules from mice. (**F**) Dynamic changes in the mean fluorescence intensity of pulmonary nodules in mice (*n* = 5, Student’s *t*-test). (**G**) Immunohistochemical staining analysis of Ki-67 levels in pulmonary nodules (*n* = 3, Student’s *t*-test). Each data point represents the mean ± SD. (* *P* < 0.05; *** *P* < 0.001)

Osteosarcoma is highly prone to lung metastasis, which remains the major cause of treatment failure and mortality [57]. Given its immunomodulatory, anti-angiogenic, and systemic anti-tumor properties, we hypothesized that OP@NPs could suppress osteosarcoma lung metastasis. To independently assess the capacity of OP@NPs to inhibit lung colonization of circulating osteosarcoma cells without confounding signals from primary bone lesions, we constructed a tail vein injection pulmonary metastasis model to mimic the hematogenous dissemination stage of osteosarcoma (Fig. 9A). Bioluminescence imaging revealed a reduction in pulmonary metastatic burden in OP@NPs-treated mice, accompanied by decreased fluorescence intensity relative to the control group (Fig. 9B and F). Gross examination of lung tissues further confirmed fewer metastatic nodules in the OP@NPs group (Fig. 9C). Histological analysis by H&E staining demonstrated substantially reduced metastatic infiltration in lung tissues following OP@NPs treatment (Fig. 9D). Ki-67 immunohistochemical analysis revealed reduced proliferative activity in metastatic lesions in the OP@NPs group (Fig. 9E and G). Although mouse models cannot completely mimic the complexity of human osteosarcoma, the intravenous metastasis model captured key features of hematogenous dissemination and lung colonization, providing a relevant platform to evaluate anti-metastatic efficacy [58]. Notably, this model only isolates the lung colonization stage and bypasses the early steps of primary tumor local invasion and spontaneous tumor cell intravasation. Therefore, tibial orthotopic spontaneous metastasis models will be adopted in our follow-up work to better mimic the full clinical metastatic progression of osteosarcoma.

## Conclusion

In this study, we constructed an OP@NPs-based nanocomposite hydrogel platform for synergistic osteosarcoma therapy. The system enabled pH-responsive and TME-activated drug release, ensuring targeted and long-term drug delivery. Combining photothermal with photodynamic therapy, OP@NPs realized effective tumor eradication. Mechanistic analysis revealed that OP@NPs exerted comprehensive anti-tumor effects via regulating cell metabolism, inducing tumor vascular normalization, and enhancing antitumor immune responses. These synergistic effects collectively inhibited tumor growth, invasion, and metastasis. Importantly, the platform has shown satisfactory therapeutic efficacy and favorable biosafety in preclinical models, indicating its potential for further translational research. Nevertheless, multiple hurdles hindered the clinical translation of this hydrogel platform. Long-term systemic toxicity of the dual-drug nanocarrier demanded extended safety evaluation in large animals. While our mild aqueous synthesis allowed lab-scale scaling, uniform mass-production workflows and cost regulation needed further refinement for industrial manufacturing. In addition, interspecies gaps between mouse orthotopic osteosarcoma models and human lesions limited direct clinical extrapolation, and subtype-tailored dosing regimens are yet to be developed. Future efforts will focus on formula optimization to mitigate systemic adverse effects, standardized scalable fabrication, and translational studies in advanced animal models to advance this combinatorial hydrogel toward clinical practice.

## Materials and methods

### Materials

Fetal bovine serum albumin (FBS), Ham’s F 12 nutrient medium and Dulbecco’s Modified Eagle Medium (DMEM) was acquired from Thermo Fisher Scientific. Table S1 lists the product codes and suppliers for all commercial flow cytometry used in this study. Table S2 lists the product codes and suppliers for all commercial antibodies used in this study. In immunoblotting, immunofluorescence, and immunohistochemistry, the antibody dilution ratios are 1:1,000, 1:200, and 1:200, respectively. Tables S3 and S4 list the product codes and suppliers for all commercial assay packages and chemical reagents used in this study.

### Preparation of hydrogel

The synthesis of oxidized sodium alginate (OSA) was carried out using a classical periodate oxidation method. In this process, 5 g of sodium alginate was homogeneously dispersed in 25 mL of ethanol to yield a uniform suspension. Meanwhile, 2.5 g of sodium periodate was dissolved in 25 mL of ultrapure water to formulate the oxidant solution. Under dark conditions, the periodate solution was slowly added dropwise into the alginate suspension, and the oxidation reaction was then allowed to proceed at room temperature for 6 h under continuous stirring. The reaction was terminated by adding 1 mL of ethylene glycol, followed by an additional 30 min of stirring to ensure complete quenching. For product isolation, the mixture was filtered with the assistance of 1 g of sodium chloride and 200 mL of ethanol. The collected solid precipitate was redissolved in ultrapure water and dialyzed against ultrapure water using a dialysis membrane with a molecular weight cutoff of 8000–14,000 Da for 2–3 days. Finally, the solid OSA product was obtained via freeze-drying.

Subsequently, for the preparation of the hydrogel (OP), 50 mg/mL OSA was fully dissolved in ultrapure water to form a homogeneous solution. Under constant mechanical stirring at 300 rpm, an aqueous solution of 50 mg/mL polyethyleneimine (PEI) was slowly added dropwise to the OSA solution. Cross-linking was achieved via a typical Schiff base reaction between the aldehyde groups on OSA chains and the amine groups on PEI molecules. This reaction entailed the condensation of carbonyl and amine groups to form imine bonds, enabling rapid cross-linking and generating hydrogel OP with a three-dimensional network structure.

### Preparation of NPs

Synthesis of D780: The synthesis process was initiated with the preparation of IR780-OH: Specifically, 3-Aminopropanol (18.2 µL, 0.24 mmol) and IR780 (80 mg, 0.12 mmol) were dissolved in anhydrous *N*,*N*-dimethylformamide (DMF, 5 mL), followed by the addition of *N*,*N*-diisopropylethylamine (DIPEA, 42 µL). Under a nitrogen atmosphere, the reaction mixture was immersed in an 85 °C oil bath with continuous stirring in the dark for 4 h. After the reaction finished, the solvent was evaporated under vacuum, and the crude product was purified by silica gel column chromatography to afford IR780-OH. The chemical identity of the product was subsequently verified by ¹H NMR spectroscopy (Bruker Avance, 400 MHz).

Subsequently, D780 was synthesized via esterification: Diclofenac (59.2 mg, 0.2 mmol) was dissolved in anhydrous DMF (5 mL), followed by the sequential addition of 4-dimethylaminopyridine (DMAP, 6 mg), 1-Ethyl-3-(3-dimethylaminopropyl) carbodiimide (EDC, 38 mg), and N-hydroxysuccinimide (NHS, 22 mg). The reaction mixture was stirred in an ice bath for 30 min to activate the system, followed by incubation at room temperature for 4 h. Thereafter, To the mixture, IR780-OH (70 mg, 0.1 mmol) was added, followed by stirring under dark conditions at room temperature for 48 h under a nitrogen atmosphere to yield the crude product. The crude product was isolated by vacuum evaporation and then chromatographed on silica gel to yield D780. Finally, the structure of the resultant compound was confirmed by high-resolution mass spectrometry (HRMS), and its purity was verified.

Preparation of NIB-Zn-D780 NPs and OP@NIB-Zn-D780 NPs: NIB-Zn-D780 nanoparticles (NPs) were synthesized via a sequential coordination and self-assembly process. Specifically, NIB (54 mg, 0.1 mmol) was fully dissolved in a mixed solvent of 1mL deionized water and 1 mL anhydrous ethanol. Subsequently, an aqueous solution of Zn^2+^ nitrate hexahydrate (Zn(NO₃)₂·6 H₂O, 30 mg, 0.1 mmol) — prepared by dissolving the salt in 1 mL deionized water—was introduced into the NIB solution, and the resulting blend was agitated overnight to allow metal-organic complex assembly. Subsequently, D780 (98 mg, 0.1 mmol), pre-dissolved in 1 mL anhydrous ethanol, was incorporated into the reaction system. The entire process was performed under a nitrogen atmosphere and in the dark to preclude photodegradation and ensure reaction stability. Following 2 h of continuous stirring, the organic solvent was removed via vacuum concentration. The resulting aqueous nanoparticle suspension was then isolated and freeze-dried to obtain a purified NIB-Zn-D780 NPs solid powder ultimately.

During the construction of the drug-loaded hydrogel system OP@NIB-Zn-D780 NPs, hereinafter referred to as OP@NPs, the synthesized NIB-Zn-D780 NPs were homogeneously dispersed in an aqueous solution of OSA via gentle stirring. Subsequently, an aqueous solution of PEI was added dropwise. The aldehyde groups of OSA readily reacted with the amine groups of PEI to form Schiff bases, thereby triggering a cross-linking reaction. Eventually, a homogeneous nanoparticle-loaded hydrogel composite, OP@NPs, was successfully obtained.

### Characterization of OP@NPs hydrogel

The morphology and microstructure of the NPs encapsulated within the hydrogel were characterized using transmission electron microscopy (TEM). A Malvern Zeta Sizer Nano analyzer was employed to evaluate the particle size distribution and dispersion stability by determining the hydrodynamic diameter and zeta potential. The three-dimensional porous structure of the OP@NPs hydrogel was visualized using SEM (scanning electron microscopy). FTIR (Fourier transform infrared) spectroscopy was utilized to identify specific functional groups in the hydrogel network through the detection of characteristic absorption peaks. Additionally, the mechanical properties and rheological behavior of the hydrogel were investigated using a rheometer.

### In vitro photothermal performance

To determine the photothermal conversion efficiency of NPs, a 1 mL NPs solution with an appropriate concentration was prepared as the test sample. This sample was subsequently irradiated with a 660 nm laser at a preset power density of 1.0 Wcm^−^². During the entire irradiation process, the solution temperature was monitored in real time using a digital thermometer, with data recorded at specified time intervals. Finally, the photothermal conversion efficiency (η) of the nanoparticles was calculated using the following formula:

$$\:\eta\:\left(\%\right)=\frac{hs{\Delta\:}{T}_{\mathrm{max}}-{Q}_{\mathrm{Dis}}}{I(1-{10}^{-A\lambda\:})}$$

$$\:{h}_{s}=\frac{mc}{{\tau\:}_{s}}$$

$$\:{\tau\:}_{s}=-\frac{t}{\mathrm{l}n\theta\:}$$

$$\:\theta\:=\frac{T-{T}_{\mathrm{surr}}}{{\Delta\:}{T}_{\mathrm{max}}}$$

For the formula presented above, each parameter is defined as follows: “h” stands for the heat transfer coefficient; “S” indicates the surface area of the container holding the solution; “ΔTmax” describes to the temperature difference between the system’s maximum achieved temperature and the ambient temperature; “Q_Dis_” represents the amount of laser energy dissipated through the solvent medium; “I” corresponds to the actual output power of the laser; and “A_λ_” is the absorbance of the nanoparticle solution at the designated detection wavelength.

### Release profile of NIB and D780 from OP@NPs

The release kinetics of NIB and D780 from OP@NPs were evaluated via dialysis under two simulated conditions: physiological environment (pH 7.4) and a tumor microenvironment (pH 5.0). Specifically, 5 mL of the OP@NPs solution was introduced into a pre-moistened dialysis bag with a molecular weight cutoff (MWCO) of 3 kDa. The dialysis bag was then immersed in phosphate-buffered saline (PBS) at the aforementioned two pH values and incubated in a constant-temperature shaking incubator at 37 °C.

At predetermined time points (0, 10, 20, 40, 60, 80, 100, and 120 h), 100 µL of external dialysate was collected. After sampling, an equal volume of fresh PBS pre-warmed to the corresponding pH was immediately supplemented to the dialysate to maintain a constant volume. All samples were subjected to quantitative analysis using a multifunctional microplate reader to determine the concentrations of released NIB and D780.

### Swelling and degradation behavior of hydrogels

*Swelling Behavior of Hydrogels*: To evaluate the swelling properties of OP@NPs hydrogels, the samples were weighed and immersed in PBS (37 °C) until equilibrium swelling was reached. After removing surface moisture, the swollen hydrogels were weighed. To obtain the swelling ratio, the following equation was employed:$\:\mathrm{Swelling\:ratio}\left(\%\right)=\frac{{W}_{t}-{W}_{0}}{{W}_{0}}\times\:100\%$

where W0 stands for the hydrogel’s initial mass, and *W*_*t*_ is the weight at time t.

*Degradation Behavior of Hydrogels*: To investigate the degradation behavior, lyophilized hydrogels were weighed (*W*_*0*_) and incubated in PBS (37 °C )with shaking at 90 rpm. The remaining weight *W*_*dt*_ was recorded at predetermined intervals. The degradation rate was calculated as:$\:\mathrm{Degradation\:rate}\left(\%\right)=\frac{{W}_{0}-{W}_{\mathrm{dt}}}{{W}_{0}}\times\:100\%$

### Cell culture

We obtained K7M2 murine osteosarcoma cells, MG63 human osteosarcoma cells, and HUVEC from ATCC (Manassas, VA, USA). The culture protocol is outlined below:

(1) K7M2 and MG63 cells were cultured in DMEM.
(2) HUVEC cells were grown in F12/DMEM.
(3) All cell lines were supplemented with 10% FBS (HyClone, SH30088.03),

streptomycin (Invitrogen), and 100 U/ml penicillin.

4. The cultures were kept in an incubator with humidity set at a temperature of 37 °C.

and a CO_2_ atmosphere of 5%.

Cellular Uptake Assay of OP@NPs.

A density of 2 × 10⁵ cells per well was used to seed MG63 and K7M2 cells in 6-well plates. When the cells reached approximately 80% confluency, the cells were exposed to culture medium supplemented with 0.2 µM or 1 µM D780, NPs, or OP@NPs, followed by incubation for predetermined durations (0, 6, 12, and 24 h). Cells treated with an equal volume of PBS at 0 h served as the control group. After incubation, adherent cells were detached with trypsin to form single-cell suspensions, which were then collected by centrifugation at 800 rpm for 3 min. The cell pellets were rinsed with pre-cooled PBS to eliminate any residual medium, resuspended in 100 µL PBS, and analyzed by flow cytometry using a BD FACSCelesta flow cytometer (USA). To ensure statistical validity, a minimum of 1 × 10⁴ cells were counted per sample. For fluorescence imaging assays, cells were treated with the same nanoparticles under identical experimental conditions. After incubation, the cells were rinsed three times with PBS (5 min per wash) to remove unbound nanoparticles. Subsequently, the cells were fixed with 4% paraformaldehyde at room temperature for 15 min. Finally, intracellular nanoparticle distribution was visualized, and micrographs were obtained via an inverted fluorescence microscope (Nikon, Tokyo, Japan).

### MTT assay

Cell viability was assessed using the MTT assay. Cells were seeded at approximately 3 × 10³ cells per well in a 96-well plate and maintained for 24 h. Subsequently, exposure to varying concentrations of NIB, NIB-Zn, D780, NPs, and OP@NPs for 12 h. Following this, the supernatant was removed, cells were gently rinsed with PBS, and then subjected to either laser irradiation (660 nm, 1.0 W cm^−^², 30s) or non-irradiation treatment. Cells were cultured for a further 12 h, after which 10 µL of MTT solution (5 mg/mL) was added. The mixture was incubated for 2 h, the culture medium removed, followed by the addition of 150 µL of dimethyl sulfoxide (DMSO). Subsequently, the optical density was recorded at 570 nm.

### Colony formation assay

A colony formation assay was performed to evaluate the long-term antiproliferative actions of the tested agents. We plated cells into 24-well plates at 5 × 10² cells per well and maintained for roughly 5 days to allow for colony initiation. The cells were then treated with NIB, NIB-Zn, D780, NPs, or OP@NPs for 12 h. After treatment, the supernatant was discarded, and the cells were rinsed twice with PBS to remove residual compounds. A portion of the samples underwent 30s (1.0 W cm^−^²) of 660 nm laser irradiation, whilst the remaining control group remained unirradiated. The culture medium was replaced every three days throughout the experiment to ensure optimal growth conditions. After a total culture period of 10 days, the cell colonies were stained with crystal violet for 30 min, gently washed three times with PBS to remove excess stain, and then fixed with 4% paraformaldehyde for 5 min. Colony images were acquired using a microscope for subsequent analysis.

### EdU labelling assay

Cell proliferation was assessed employing the EdU labelling assay. Cells were plated into 96-well plates and subjected to treatment as described. After removing the supernatant, the cells were treated with or without 660 nm laser irradiation (1.0 W cm^−^², 30 s) and then cultured for an additional 12 h. Utilizing the Kit (Ribobio, C10310-3), the proportion of continuously replicating cells was determined.

### Apoptosis assay

Annexin V-FITC/PI double staining followed by flow cytometry was employed to evaluate apoptosis. We placed 5 × 10⁴ cells per well into 6-well plates and incubated them for 24 h. Next, the cells were then treated with NIB, NIB-Zn, D780, NPs, or OP@NPs for 12 h. Subsequently, the cells underwent laser irradiation (660 nm, 1.0 W cm^−^², 30s). This is followed by another 12-hour incubation period. Lastly, the cells were subjected and co-staining with Annexin V-FITC and propidium iodide (PI) at 4 °C for 15 min. Flow cytometric detection of apoptotic cells was performed per the manufacturer’s guidelines.

### Evaluation of mitochondrial membrane potential

For mitochondrial membrane potential measurement, we performed the JC-1 staining method. 6-well plates received 5 × 10⁴ cells per well, then were left undisturbed overnight for cell attachment. Subsequently, the cells were treated with the specified drugs for 12 h. Subsequently, the cells underwent laser irradiation (660 nm, 1.0 W cm^−^², 30s) and then cultured for an additional 12 h. The mitochondrial membrane was stained using the JC-1 solution for a duration of 20 min. The mitochondrial membrane potential was then quantified using a flow cytometer as per the manufacturer’s guidelines.

### Cell live/dead staining assay

24-well plates were seeded with cells and kept for one day. The next step involved treatment with NIB, NIB-Zn, D780, NPs, and OP@NPs for 12 h. Subsequently, the cells underwent laser irradiation (660 nm, 1.0 W cm^−^², 30s), and then cultured for an additional 12 h. The cells were co-stained with Calcein-AM and PI. The stained cells were then incubated in the dark at 37 °C under 5% CO₂ for 30 min. Finally, according to the manufacturer’s instructions, fluorescence microscopy was employed to capture images and evaluate cell viability (live/dead status).

### In VItro ROS generation

Cells were seeded into 6-well plates and incubated for 24 h. Subsequently, they were treated with the specified drugs for an additional 12 h. Subsequently, the cells underwent laser irradiation (660 nm, 1.0 W cm^−^², 30s), and then cultured for an additional 12 h. Following treatment, the cells were rinsed twice with PBS to remove residual medium and drugs, and then collected by centrifugation at a speed of 1000 rpm. The pellet was resuspended in serum-free medium, and the DCFH-DA probe was added. The mixture was incubated at 37 °C for 30 min for probe loading. Post-incubation, unbound probe was removed by three PBS washes. Fluorescence intensity was measured using a FACSCalibur flow cytometer, and data analysis was carried out using the FlowJo VX software.

### Intracellular ATP content detection

We seeded cells in 6-well plates and allowed them to grow for 24 h. Following this, the cells were treated with NIB, NIB-Zn, D780, NPs, and OP@NPs for 12 h. Subsequently, the cells underwent laser irradiation (660 nm, 1.0 W cm^−^², 30s), and then cultured for an additional 12 h. Once the post-irradiation incubation finished, the cells were washed three times with PBS to eliminate residual reagents and culture medium. The intracellular ATP content was then measured following the instructions supplied with the ATP detection kit.

### Lactic acid detection assay

Cells were seeded in 6-well plates and cultured for 24 h to allow for adherent growth. Subsequently, the cells were treated with NIB, NIB-Zn, D780, NPs, and OP@NPs for 12 h. Subsequently, the cells underwent laser irradiation (660 nm, 1.0 W cm^−^², 30s), and then cultured for an additional 12 h. At the end of incubation, the cell culture medium was carefully aspirated. The extracellular lactic acid content was then measured following the manufacturer’s instructions for the lactic acid detection kit.

### Extracellular Acidification Rate (ECAR) detection assay

We seeded 2 × 10⁴ cells per well in 96-well plates and cultured overnight. Following the removal of culture medium, 100 µL of detection reagent was added to each well, followed by treatment with the respective drugs (NIB, NIB-Zn, D780, NPs, and OP@NPs) for 12 h. Subsequently, the cells underwent laser irradiation (660 nm, 1.0 W cm^−^², 30s). After irradiation, fluorescence intensity at 535 nm was measured every 5–10 min over a 120-minute timeframe, following the manufacturer’s instructions. The extracellular acidification rate (ECAR) was then computed based on the dynamic changes in fluorescence intensity throughout the detection process.

### Glucose uptake fluorescence detection assay

Cells were seeded in 6-well plates and incubated overnight. Once the culture medium was discarded, PBS was used to wash the cells one time. The cells were then cultured in glucose-free medium (supplemented with serum) for 6 h to induce glucose depletion. After glucose depletion, the cells were treated with drugs (NIB, NIB-Zn, D780, NPs, and OP@NPs) diluted in glucose-free medium, followed by 660 nm laser irradiation (1 W cm^−^², 30s). The cells were incubated for an additional 12 h after irradiation. At the end of incubation, the cells were washed twice with PBS, digested, and centrifuged at 4000 rpm for 3 min to collect the pellet. The pellet was resuspended in serum-free medium, and the 2-NBDG probe was added. The mixture was incubated at 37 °C for 30 min to load the probe. Once incubation was complete, three PBS washes were performed on the cells to clear away excess probe. Finally, glucose uptake was measured by fluorescence intensity using a FACSCalibur flow cytometer. Data were analyzed with Flowjo VX software to quantify the glucose uptake levels of each experimental group.

### Pyruvic acid rescue experiment

Cells were seeded in 6-well plates and cultured for 24 h to achieve complete adherence. Four groups were established: NC, pyruvic acid (PA), OP@NPs, and PA + OP@NPs. Pyruvic acid was applied at a final concentration of 1 mM, which is widely adopted for in vitro cellular metabolic rescue assays. Cells received 12 h incubation with different prepared formulations. After treatment, cell culture supernatants were harvested for the quantification of extracellular lactic acid levels based on the specifications of the commercial detection kit. Cell viability was further evaluated via live/dead fluorescent staining. Cells were washed twice with PBS and incubated with live/dead staining solution at 37 °C for 20 min in the dark. After removing excess staining solution via PBS rinsing, fluorescence images were captured using an inverted fluorescence microscope for subsequent analysis.

### Immunoblotting

The cells were seeded at 5 × 10⁴ cells per well in six-well plates and cultured for 24 h. Next, the cells were subjected to the specified treatment. Following that, the cells were washed with pre-chilled PBS and lysed using radioimmunoprecipitation assay (RIPA) buffer, with sonication to aid lysis. The harvested lysates were resolved by SDS-PAGE, then electrotransferred to PVDF membranes. Blocking was carried out with skim milk at ambient temperature for 2 h, followed by overnight incubation at 4 °C with moderate agitation. the specified primary antibodies were subjected to further treatment by the application of secondary antibodies for an extra 2 h. Protein expression was assessed using the ChemiScope 6000 Touch instrument (Clinx, Shanghai) following incubation with Immobilon Western HRP Substrate (Millipore, WBKLS0050).

### Wound healing assay

Cells were plated in 6-well plates at 5 × 10⁵ per well and exposed to the treatments outlined above. After treatment, the cells were cultured until 90–100% confluency. Three parallel scratches were made in each well using the tip of a 200 µL pipette. The cells were then rinsed 2–3 times with PBS to remove detached debris, and serum-free medium was added to start the wound healing process. Images of the scratch areas were captured at 0, 12, and 36 h using an inverted fluorescence microscope (Nikon, Tokyo, Japan). The cell migration rate was calculated using the gap area method in the ImageJ software.

### Transwell migration assay

We seeded 5 × 10⁵ cells per well in 6-well plates. Once 90%–100% confluency was reached, the cells were serum-starved for 24 h, followed by drug treatment as described above. Following drug exposure, the cells received a PBS wash and were then re-suspended in serum-free medium to reach 2 × 10⁵ cells. These cells were subsequently placed into the upper Transwell chambers at the indicated density per well. The upper chambers were placed into a 24-well plate containing 600 µL complete medium. After 24 h of incubation, the chambers were transferred to a new 24-well plate with 600 µL 4% paraformaldehyde and incubated for 30 min. After removing non-migrated cells, the upper chambers were stained with 600 µL crystal violet solution for 10 min. Stained cells were observed and photographed using an Olympus SZX16 stereomicroscope, and cell counting was performed using ImageJ software.

### Transwell invasion assay

A density of 5 × 10⁵ cells per well was used to seed 6-well plates. Once 90%–100% confluency was reached, the cells were serum-starved for 24 h. During this time, 25 µL of 10% matrix (v/v; matrix: DMEM = 1:9) was evenly coated on the bottom of the upper chambers of Transwell inserts and incubated at 37 °C for preparation. After serum starvation, the cells were treated with drugs as described previously. Following drug treatment, the cells were washed with PBS, resuspended in serum-free medium at a concentration of 2 × 10⁵ cells per well, and seeded into the pre-coated upper chambers of Transwell inserts. The upper chambers were placed into a 24-well plate containing 600 µL of complete medium for incubation. After 24 h, the upper chambers were transferred to a new 24-well plate containing 600 µL of 4% paraformaldehyde and incubated for 30 min. After removing non-invaded cells, the upper chambers were stained with 600 µL of crystal violet solution for 10 min. Stained cells were observed and photographed using an Olympus SZX16 stereomicroscope, and cell counting was performed using ImageJ software.

### Tube formation assay

HUVEC cells were plated at 5 × 10⁵ per well into 6-well plates and incubated for 24 h. After 24 h, the cells were treated with NIB, NIB-Zn, D780, NPs, and OP@NPs for 12 h, followed by drug treatment as described above. Meanwhile, 50 µL of Matrigel was added to each well of a 24-well plate and evenly spread across the bottom of the well, then placed at 4 °C overnight to solidify. After drug treatment, the cells were collected and seeded into pre-coated 24-well plates at a density of 1 × 10⁵ cells per well. To each well, 200 µL of culture medium was added, and the cells were incubated for an additional 24–48 h. After incubation, tube formation was observed and photographed using an inverted fluorescence microscope (Nikon, Tokyo, Japan). The tube formation was then analyzed using ImageJ software.

### Plug assay

Female BALB/c mice (6 weeks old) were randomly divided into 6 groups (*n* = 3): control, NIB, NIB-Zn, D780, NPs, and OP@NPs. Matrigel was taken out from the − 20 °C freezer and thawed at 4 °C for 12 h until it became liquid. Mice in the control group were subcutaneously injected with 0.5 mL of pure Matrigel near the abdominal midline. Mice in the other groups were subcutaneously injected with 0.5 mL of Matrigel containing the corresponding drug (dose: 2 mg/kg) at the same injection site. After 2 weeks of feeding, the mice were euthanized, and the subcutaneous Matrigel plugs were excised entirely and photographed for documentation. Hemoglobin concentration in the Matrigel plugs was then measured using Drabkin’s reagent, strictly following the kit instructions, to evaluate angiogenesis.

### Impact of lactate metabolism on osteosarcoma immune microenvironment

To investigate the role of lactate metabolism in modulating the osteosarcoma immune microenvironment, we performed the following bioinformatics analysis: The osteosarcoma transcriptome dataset GSE42352 from the GEO database was integrated, and batch effects were removed using standardization to guarantee data quality and comparability. Lactate metabolism-associated genes (LAGs) were screened from the Gene Ontology (GO) database, including GO_LACTATE_METABOLIC_PROCESS and GO_LACTATE_TRANSPORT. The expression profiles of these genes were analyzed, and osteosarcoma samples in the GSE42352 dataset were divided into two groups (Cluster 1 and Cluster 2) based on lactate metabolism characteristics via consensus clustering. Lastly, the CIBERSORT tool was applied to quantify the relative infiltration of immune cells, including T cells, macrophages, NK cells. The influence of lactate metabolism on the immune cell composition and distribution in the osteosarcoma immune microenvironment was analyzed through inter-group comparison, offering data support for further mechanistic investigations.

### Co-culture assay

We seeded K7M2 cells in 6-well plates and allowed them to grow for 24 h. The cells were then treated with NIB, NIB-Zn, D780, NPs, and OP@NPs, followed by drug treatment as described above. Upon completion of the post-irradiation incubation, the treated K7M2 cells were reseeded into the upper chamber of Transwell inserts. Meanwhile, immune cells isolated from 6-week-old female BALB/c mice were seeded into the lower chamber of the transwell inserts and co-cultured for 48 h. Following co-culture, immune cells from the lower chamber were collected, and phenotypic characteristics and proportions of immune cells were analyzed using a FACSCalibur flow cytometer.

### Enzyme-Linked Immunosorbent Assay (ELISA)

The levels of TGF-β, TNF-α, IL-2, IFN-γ, and Granzyme B in the co-culture supernatant or mouse serum were measured using BOSTER enzyme-linked immunosorbent assay (ELISA) kits. The assays were performed according to the manufacturer’s instructions, allowing for the quantitative analysis of these immune factors to assess immune response and inflammation levels.

### In vivo photothermal effect and metabolism of OP@NPs

To systematically evaluate the in vivo functional properties of OP@NPs, studies on their in vivo photothermal effect and metabolic process were conducted as follows: In Vivo Photothermal Effect Detection: NPs or OP@NPs (volume: 100 µL, dosage: 2 mg/kg) were injected into the back of Balb/c mice. Immediately after injection, the mice were irradiated with a 660 nm laser (1 W cm^−^²). Under the anesthetized state of the mice, an infrared thermal imager (UNI-T) was used to record the temperature change of the injection area at specified time points (0, 1, 2, 3, 4 min) to analyze the photothermal conversion efficiency of OP@NPs. In Vivo Metabolism Study: Using the same injection protocol (NPs or OP@NPs, 100 µL, 2 mg/kg, injected into the back of the mice), under the anesthetized state of the mice, in vivo imaging was performed with a small animal in vivo imager (AnIView Kirin) at specified time points (1, 2, 4, 6, 8, 10, 12, 14 days). The metabolic process and clearance of OP@NPs in vivo were analyzed by monitoring the dynamic change of fluorescence signal intensity in the injection area.

### Establishment of in situ MG63 and subcutaneous K7M2 tumor models

An orthotopic MG63-OS xenograft model was established using 6-week-old Balb/c nude mice. MG63 cells were resuspended in PBS at 4 °C for later use. General anesthesia with isoflurane and local periosteal anesthesia with 5% lidocaine were applied. The surgical area of the right hind limb was disinfected with povidone-iodine, and the knee joint was flexed to more than 90°. A 29-G sterile needle was used to penetrate the proximal tibial cortex, and 1 × 10⁶ MG63 cells were injected into the tibial medullary cavity under anesthesia. After surgery, mice were placed under a heating lamp until they regained consciousness. A subcutaneous K7M2-OS xenograft model was established using 6-week-old Balb/c mice. K7M2 cells were resuspended in PBS at 4 °C for later use. After anesthesia with isoflurane, 1 × 10⁶ K7M2 cells were injected subcutaneously into the inguinal region using a 29G sterile needle. Tumor growth was monitored, and once the primary tumor volume reached approximately 75 mm³, the tumor-bearing mice were used for subsequent in vivo experiments.

### In vivo efficacy evaluation of OS models

In vivo efficacy was evaluated in the MG63 osteosarcoma (OS) orthotopic xenograft and K7M2 OS subcutaneous xenograft models as follows: MG63 OS model mice were randomly divided into 4 groups (5 mice per group), and K7M2 OS model mice into 6 groups (5 mice per group), both receiving peritumoral injections. The MG63 OS groups received PBS, NIB-Zn, NPs, and OP@NPs; the K7M2 OS groups received PBS, NIB, NIB-Zn, D780, NPs, and OP@NPs. The dosages were NIB 30 mg/kg, D780 2 mg/kg, with consistent drug concentrations across groups. All mice were subjected to 30-second laser irradiation 24 h after drug injection. Starting from the first dose, tumor volume and body weight were assessed at 3-day intervals, with a monitoring period of 21 days. Tumor volume (V) was calculated using the formula V (mm³) = length × width² × 0.5. After the monitoring period, all mice were euthanized, and tumor tissues were excised, weighed, and photographed. The tissues were then sent for histological analysis.

### Evaluation of anti-metastatic effects in vivo

To assess the anti-metastatic effect of the target preparations, a mouse lung metastasis model was established via tail vein injection. 1 × 10⁶ K7M2 cells were injected into mice through the tail vein. Once the model was successfully established, the tumor-bearing mice were randomly divided into groups and treated according to the previously described in vivo treatment protocol (peritumoral subcutaneous injection, once a week; the control group received 100 µL of normal saline, and the experimental groups received preparations containing NIB, NIB-Zn, D780, NPs, and OP@NPs, with NIB dosed at 30 mg/kg and D780 at 2 mg/kg; 30-second laser irradiation was performed 24 h post-administration). During the treatment period, in vivo imaging was performed on days 10, 13, 17, and 20 using a small animal in vivo imager (AnIView Kirin) to dynamically observe lung metastasis. After the monitoring period, all mice were euthanized, and their lung tissues were completely excised and photographed for documentation. The lung tissues were then fixed in Bouin’s solution, and metastatic nodules in the lungs were displayed and counted using the fixed tissue samples. Finally, the fixed lung tissues were sent for subsequent histological analysis.

### In vivo evaluation of OP@NPs combined with α-PD-1 for osteosarcoma treatment

Using the K7M2 OS subcutaneous xenograft model, the in vivo efficacy of OP@NPs combined with α-PD-1 (anti-mouse PD-1 antibody: MedChemExpress, Cat. No. HY-P99144) was evaluated as follows: K7M2 OS model mice were randomly divided into 4 groups (5 mice per group). The administration protocol was as follows: the control group received PBS; the α-PD-1 group was injected with α-PD-1 via the tail vein; the OP@NPs group was injected with OP@NPs; the combined treatment group received both OP@NPs and α-PD-1 via the tail vein. The dosages were 10 mg/kg for α-PD-1 and 2 mg/kg for OP@NPs. Mice injected with OP@NPs (including the OP@NPs and combined treatment groups) received 30 s laser irradiation 24 h after injection. Tumor volume and body weight were measured every 3 days starting from the first administration, with a monitoring period of 21 days. Tumor volume (V) was calculated using the formula V (mm³) = length × width² × 0.5. After the monitoring period, all mice were euthanized, and tumor tissues were excised, weighed, and photographed. The tissues were then sent for histological analysis.

### In vivo DC101 blocking angiogenesis assay

In vivo anti-angiogenic efficacy was evaluated in the K7M2 osteosarcoma subcutaneous xenograft model. Model mice were randomly divided into 4 groups (5 mice per group): NC, anti-mouse VEGFR-2 antibody DC101, OP@NPs, and DC101 + OP@NPs combination group. DC101 was administered via intraperitoneal injection at a dosage of 10 mg/kg, while OP@NPs was given by peritumoral injection with the consistent dosage described in the above in vivo efficacy evaluation. For the combination group, DC101 was intraperitoneally injected in advance to block tumor angiogenesis before OP@NPs peritumoral administration. All mice were subjected to 30 s laser irradiation (660 nm, 1.0 W·cm⁻²) 24 h after OP@NPs injection. Starting from the first dose, tumor volume and body weight were assessed at 3-day intervals, with a monitoring period of 21 days. Tumor volume (V) was calculated using the formula V (mm³) = length × width² × 0.5. After the monitoring period, all mice were euthanized, and tumor tissues were excised, weighed, and photographed. The tissues were then collected for subsequent histological and angiogenesis-related immunohistochemical analysis.

### Safety evaluation

The heart, lungs, liver, spleen, and kidneys were collected and rinsed with pre-chilled PBS to remove red blood cells. The tissues were fixed in 4% formaldehyde for 48 h, then embedded in paraffin and stained with HE. For hematological analysis, blood was collected from the orbital venous plexus, allowed to clot overnight, and 150 µL of serum was used to measure AST, ALT, BUN, CKMB2, UREAL, and creatinine (Cre) levels.

### Immunohistochemistry

Tumor xenografts were fixed in formalin and paraffin-embedded, then sectioned onto pre-adhered slides. Primary antibodies were incubated overnight on the slides. Following secondary antibody incubation, sections were stained using 3,3’-diaminobenzidine (DAB) according to established protocols. All images were captured using a DM2500 fluorescence microscope (Danaher Corporation, Wetzlar, Germany).

### Statistical analysis

All statistical evaluations and data visualization were carried out using GraphPad 10 software (GraphPad, USA). The statistical methods were as follows: One-way ANOVA for comparisons among three or more samples, Student’s *t*-test for comparisons between two samples, and Two-way ANOVA for comparisons among three or more samples involving two factors. Each experiment was conducted a minimum of three independent times, with data presented as “mean ± SD”. Significance levels were established by as follows: *P < 0.05 indicates significance, **P < 0.01 indicates high significance, and ***P < 0.001 indicates extremely high significance.

## Supplementary Information

Supplementary Material 1

## Data Availability

All data included in this study are available upon reasonable request of corresponding authors.

## References

[1] Gill J, Gorlick R. Advancing therapy for osteosarcoma. Nat Rev Clin Oncol. 2021;18(10):609–24. 34131316 10.1038/s41571-021-00519-8

[2] Bishop M, Janeway K, Gorlick R. Future directions in the treatment of osteosarcoma. Curr Opin Pediatr. 2016;28(1):26–33. 26626558 10.1097/MOP.0000000000000298 PMC4761449

[3] Zhu T, Han J, Yang L, Cai Z, Sun W, Hua Y, et al. Immune Microenvironment in Osteosarcoma: Components, Therapeutic Strategies and Clinical Applications. Front Immunol. 2022;13:17. 10.3389/fimmu.2022.907550 PMC9198725 35720360

[4] Chao Y, Liu Z. Biomaterials tools to modulate the tumour microenvironment in immunotherapy. Nat Rev Bioeng. 2023;1(2):125–38.

[5] Ouyang A, Qin X, Su B, Mo S, Jiang J, Wang Z, et al. Metformin glycyrrhetinic acid binary injectable hydrogel for synergistic tumor immunotherapy via spatiotemporal microenvironment remodeling. Mater Today Bio. 2026;36:102749. 41560796 10.1016/j.mtbio.2025.102749 PMC12813322

[6] Qiao E, Qiu F, Guo X, Ding J, Chen X, Zheng L, et al. Biomaterial-mediated modulation of tumor microenvironment and therapeutic sensitization. EngMedicine. 2025;2(4):100092.

[7] Szeto G, Finley S. Integrative Approaches to Cancer Immunotherapy. Trends Cancer. 2019;5(7):400–10. 31311655 10.1016/j.trecan.2019.05.010 PMC7467854

[8] Yu S, Yao X. Advances on immunotherapy for osteosarcoma. Mol Cancer. 2024;23(1):21. 39245737 10.1186/s12943-024-02105-9 PMC11382402

[9] Zhang Y, Peng Q, Zheng J, Yang Y, Zhang X, Ma A, et al. The function and mechanism of lactate and lactylation in tumor metabolism and microenvironment. Genes Dis. 2023;10(5):2029–37. 37492749 10.1016/j.gendis.2022.10.006 PMC10363641

[10] Watson M, Vignali P, Mullett S, Overacre-Delgoffe A, Peralta R, Grebinoski S, et al. Metabolic support of tumour-infiltrating regulatory T cells by lactic acid. Nature. 2021;591(7851):645–. 33589820 10.1038/s41586-020-03045-2 PMC7990682

[11] Certo M, Tsai C, Pucino V, Ho P, Mauro C. Lactate modulation of immune responses in inflammatory versus tumour microenvironments. Nat Rev Immunol. 2021;21(3):151–61. 32839570 10.1038/s41577-020-0406-2

[12] Zhang D, Tang Z, Huang H, Zhou G, Cui C, Weng Y, et al. Metabolic regulation of gene expression by histone lactylation. Nature. 2019;574(7779):575–. 31645732 10.1038/s41586-019-1678-1 PMC6818755

[13] Colbert L, El Alam M, Wang R, Karpinets T, Lo D, Lynn E, et al. Tumor-resident Lactobacillus iners confer chemoradiation resistance through lactate-induced metabolic rewiring. Cancer Cell. 2023;41(11):1945–. 37863066 10.1016/j.ccell.2023.09.012 PMC10841640

[14] Feng Y, Xiong Y, Qiao T, Li X, Jia L, Han Y. Lactate dehydrogenase A: A key player in carcinogenesis and potential target in cancer therapy. Cancer Med. 2018;7(12):6124–36. 30403008 10.1002/cam4.1820 PMC6308051

[15] Shim H, Dolde C, Lewis B, Wu C, Dang G, Jungmann R, et al. c-Myc transactivation of LDH-A: Implications for tumor metabolism and growth. Proc Natl Acad Sci U S A. 1997;94(13):6658–63. 9192621 10.1073/pnas.94.13.6658 PMC21214

[16] Dang C, Le A, Gao P. MYC-Induced Cancer Cell Energy Metabolism and Therapeutic Opportunities. Clin Cancer Res. 2009;15(21):6479–83. 19861459 10.1158/1078-0432.CCR-09-0889 PMC2783410

[17] Gottfried E, Lang SA, Renner K, Bosserhoff A, Gronwald W, Rehli M, et al. New aspects of an old drug–diclofenac targets MYC and glucose metabolism in tumor cells. PLoS ONE. 2013;8(7):e66987. 23874405 10.1371/journal.pone.0066987 PMC3706586

[18] Wang D, Dubois RN. The role of COX-2 in intestinal inflammation and colorectal cancer. Oncogene. 2010;29(6):781–8. 19946329 10.1038/onc.2009.421 PMC3181054

[19] Zhang X, Hou H, Wan J, Yang J, Tang D, Zhao D, et al. Inhibiting COX-2/PGE2 pathway with biodegradable NIR-II fluorescent polymeric nanoparticles for enhanced photodynamic immunotherapy. Nano Today. 2023;48:15.

[20] Wang Y, Xu C, Meng M, Lin L, Hu Y, Hao K, et al. Precise regulation of inflammation and immunosuppressive microenvironment for amplified photothermal/immunotherapy against tumour recurrence and metastasis. Nano Today. 2021;40:10.

[21] Tu J, Xu H, Ma L, Li C, Qin W, Chen X, et al. Nintedanib enhances the efficacy of PD-L1 blockade by upregulating MHC-I and PD-L1 expression in tumor cells. Theranostics. 2022;12(2):747–66. 34976211 10.7150/thno.65828 PMC8692903

[22] Chen J, Bu C, Lu Y, Peng X, Yu J, Ding X, et al. Bioresponsive nanoreactor initiates cascade reactions for tumor vascular normalization and lactate depletion to augment immunotherapy. Biomaterials. 2025;317:123100. 39799700 10.1016/j.biomaterials.2025.123100

[23] Tan M, Cao G, Wang R, Cheng L, Huang W, Yin Y, et al. Metal-ion-chelating phenylalanine nanostructures reverse immune dysfunction and sensitize breast tumour to immune checkpoint blockade. Nat Nanotechnol. 2024;19(12):1903–13. 39187583 10.1038/s41565-024-01758-3

[24] Nam H, Park H, Son MK, Kang I, Choi Y, Lee S, et al. Metal-phenolic networks reverse the immunosuppressive tumor microenvironment via dual metabolism regulation and immunogenic cell death. J Controlled Release. 2025;383:113775. 10.1016/j.jconrel.2025.113775 40294797

[25] Colombo G, Serganova I, Ballesio F, Kang JH, Karakousi T, Blasberg R et al. Enhancing immunotherapy efficacy in glycolytic tumors via antiangiogenic modulation. J Immunother Cancer. 2025;13(Suppl 2):A417.

[26] Zhou J, Hu Y, Cao Y, Ding S, Zeng L, Zhang Y, et al. A Lactate-Depleting metal organic framework-based nanocatalyst reinforces intratumoral T cell response to boost anti-PD1 immunotherapy. J Colloid Interface Sci. 2024;660:869–84. 38277843 10.1016/j.jcis.2024.01.129

[27] Yang L, Sun Q, Chen S, Ma D, Qi Y, Liu H, et al. pH-responsive hydrogel with gambogic acid and calcium nanowires for promoting mitochondrial apoptosis in osteosarcoma. J Controlled Release. 2025;377:563–77. 10.1016/j.jconrel.2024.11.055 39603540

[28] Li Q, Gao Y, Li R, Li H, Yang C, Li H, et al. Bioactive glass with integration of hyperthermia to manipulate pH-responsive release of chemotherapeutic agent for osteosarcoma combating and time-sequential reconstruction of bone homeostasis. Chem Eng J. 2025;524:169076.

[29] Kortam S, Lu Z, Zreiqat H. Recent advances in drug delivery systems for osteosarcoma therapy and bone regeneration. Commun Mater. 2024;5(1):168.

[30] Li J, Zhou X, Liu S, Ouyang A, Su B, Wang Z et al. Augmented Therapeutic Efficacy of Erianin through pH-Responsive Charge-Reversal Liposome Integrated Synergistic PTT and PDT in Breast Cancer. J Pharm Anal. 2026;16(7):101555. 42540442 10.1016/j.jpha.2026.101555 PMC13425661

[31] Ma J, Li P, Wang W, Wang S, Pan X, Zhang F, et al. Biodegradable Poly(amino acid)-Gold-Magnetic Complex with Efficient Endocytosis for Multimodal Imaging-Guided Chemo-photothermal Therapy. ACS Nano. 2018;12(9):9022–32. 30059614 10.1021/acsnano.8b02750

[32] Yu C, Ding B, Zhang X, Deng X, Deng K, Cheng Z, et al. Targeted iron nanoparticles with platinum-(IV) prodrugs and anti-EZH2 siRNA show great synergy in combating drug resistance in vitro and in vivo. Biomaterials. 2018;155:112–23. 29175080 10.1016/j.biomaterials.2017.11.014

[33] Li L, Tian H, Zhang Z, Ding N, He K, Lu S, et al. Carrier-Free Nanoplatform via Evoking Pyroptosis and Immune Response against Breast Cancer. ACS Appl Mater Interfaces. 2023;15(1):452–68. 36538368 10.1021/acsami.2c17579

[34] Zheng X, Carstens J, Kim J, Scheible M, Kaye J, Sugimoto H, et al. Epithelial-to-mesenchymal transition is dispensable for metastasis but induces chemoresistance in pancreatic cancer. Nature. 2015;527(7579):525–. 26560028 10.1038/nature16064 PMC4849281

[35] Nieto M, Huang R, Jackson R, Thiery J. EMT: 2016. Cell. 2016;166(1):21–45. 27368099 10.1016/j.cell.2016.06.028

[36] Llibre A, Kucuk S, Gope A, Certo M, Mauro C, Lactate. A key regulator of the immune response. Immunity. 2025;58(3):535–54. 40073846 10.1016/j.immuni.2025.02.008

[37] Xu N, Zhu Y, Han Y, Liu Q, Tong L, Li Y, et al. Targeting MondoA-TXNIP restores antitumour immunity in lactic-acid-induced immunosuppressive microenvironment. Nat Metab. 2025;7(9):28. 10.1038/s42255-025-01347-1 40846790

[38] Chen S, Xu Y, Zhuo W, Zhang L. The emerging role of lactate in tumor microenvironment and its clinical relevance. Cancer Lett. 2024;590:18. 10.1016/j.canlet.2024.216837 38548215

[39] Faubert B, Solmonson A, DeBerardinis RJ. Metabolic reprogramming and cancer progression. Science. 2020;368(6487):eaaw5473. 32273439 10.1126/science.aaw5473 PMC7227780

[40] LeBleu V, O’Connell J, Herrera K, Wikman H, Pantel K, Haigis M, et al. PGC-1α mediates mitochondrial biogenesis and oxidative phosphorylation in cancer cells to promote metastasis (16, pg 992, 2014). Nat Cell Biol. 2014;16(11):1125. 10.1038/ncb3039 PMC4369153 25241037

[41] Hanahan D, Weinberg RA. Hallmarks of cancer: the next generation. Cell. 2011;144(5):646–74. 21376230 10.1016/j.cell.2011.02.013

[42] Brand A, Singer K, Koehl G, Kolitzus M, Schoenhammer G, Thiel A, et al. LDHA-Associated Lactic Acid Production Blunts Tumor Immunosurveillance by T and NK Cells. Cell Metab. 2016;24(5):657–71. 27641098 10.1016/j.cmet.2016.08.011

[43] Kiesel VA, Sheeley MP, Coleman MF, Cotul EK, Donkin SS, Hursting SD, et al. Pyruvate carboxylase and cancer progression. Cancer Metab. 2021;9(1):20. 33931119 10.1186/s40170-021-00256-7 PMC8088034

[44] Kabir AU, Subramanian M, Kwon Y, Choi K. Linking tumour angiogenesis and tumour immunity. Nat Rev Immunol. 2026; 26(1): 35‑51. 40813802 10.1038/s41577-025-01211-z

[45] Borst J, Ahrends T, Bąbała N, Melief CJM, Kastenmüller W. CD4(+) T cell help in cancer immunology and immunotherapy. Nat Rev Immunol. 2018;18(10):635–47. 30057419 10.1038/s41577-018-0044-0

[46] Zhang L, Romero P. Metabolic Control of CD8(+) T Cell Fate Decisions and Antitumor Immunity. Trends Mol Med. 2018;24(1):30–48. 29246759 10.1016/j.molmed.2017.11.005

[47] Zhang C, Li J, Xu D, Wang D, Qi M, Chen F, et al. Surface Molecularly Engineered Mitochondria Conduct Immunophenotype Repolarization of Tumor-Associated Macrophages to Potentiate Cancer Immunotherapy. Adv Sci. 2024;11(38):18. 10.1002/advs.202403044 PMC11481252 39119940

[48] Mantovani A, Biswas S, Galdiero M, Sica A, Locati M. Macrophage plasticity and polarization in tissue repair and remodelling. J Pathol. 2013;229(2):176–85. 23096265 10.1002/path.4133

[49] Hato L, Vizcay A, Eguren I, Pérez-Gracia J, Rodríguez J, Pérez-Larraya J, et al. Dendritic Cells in Cancer Immunology and Immunotherapy. Cancers. 2024;16(5):23. 10.3390/cancers16050981 PMC10930494 38473341

[50] Pittet M, Di Pilato M, Garris C, Mempel T. Review Dendritic cells as shepherds of T cell immunity in cancer. Immunity. 2023;56(10):2218–30. 37708889 10.1016/j.immuni.2023.08.014 PMC10591862

[51] Tian L, Goldstein A, Wang H, Ching Lo H, Sun Kim I, Welte T, et al. Mutual regulation of tumour vessel normalization and immunostimulatory reprogramming. Nature. 2017;544(7649):250–4. 28371798 10.1038/nature21724 PMC5788037

[52] Gospos J, Laubach M, Savi FM, Ravichandran A, Bauer J, Friedrich O, et al. Dynamic assessment of a humanized bone tumour microenvironment reveals insights into osteosarcoma primary tumour remodelling and lung metastases. Sci Rep. 2025;15(1):45619. 41315643 10.1038/s41598-025-29941-z PMC12753712

[53] Fukurnura D, Kloepper J, Amoozgar Z, Duda D, Jain R. Enhancing cancer immunotherapy using antiangiogenics: opportunities and challenges. Nat Rev Clin Oncol. 2018;15(5):325–40. 29508855 10.1038/nrclinonc.2018.29 PMC5921900

[54] Ul Kabir A, Subramanian M, Kwon Y, Choi K. Linking tumour angiogenesis and tumour immunity. Nat Rev Immunol. 2026; 26(1): 35‑51. 10.1038/s41577-025-01211-z 40813802

[55] Jain R. Antiangiogenesis Strategies Revisited: From Starving Tumors to Alleviating Hypoxia. Cancer Cell. 2014;26(5):605–22. 25517747 10.1016/j.ccell.2014.10.006 PMC4269830

[56] Allen E, Jabouille A, Rivera L, Lodewijckx I, Missiaen R, Steri V, et al. Combined antiangiogenic and anti-PD-L1 therapy stimulates tumor immunity through HEV formation. Sci Transl Med. 2017;9(385):13. 10.1126/scitranslmed.aak9679 PMC5554432 28404866

[57] Isakoff MS, Bielack SS, Meltzer P, Gorlick R. Osteosarcoma: Current Treatment and a Collaborative Pathway to Success. J Clin Oncol. 2015;33(27):3029–35. 26304877 10.1200/JCO.2014.59.4895 PMC4979196

[58] Sunbul FS, Almuqbil RM, Zhang H, Alhudaithi SS, Fernandez ME, Aldaqqa RR, et al. An improved experimental model of osteosarcoma lung metastases to investigate innovative therapeutic interventions and sex as a biological variable. Int J Pharm. 2025;673:125372. 39971171 10.1016/j.ijpharm.2025.125372 PMC12795211

