# Supplementary material for: Phototherapy-responsive nanoplatform with hydrogel delivery potentiates immunotherapy and inhibits lung metastasis in osteosarcoma via synergistic glycolysis inhibition and vascular normalization
Source: J Nanobiotechnology. 2026 Aug 12;24:846. doi: 10.1186/s12951-026-04906-0 (PMC13551941; doi:10.1186/s12951-026-04906-0)
Supplement: Supplementary file 1 [file 12951_2026_4906_MOESM1_ESM.docx]

**Supporting Information**

**Phototherapy-Responsive Nanoplatform with Hydrogel Delivery Potentiates Immunotherapy and Inhibits Lung Metastasis in Osteosarcoma via Synergistic Glycolysis Inhibition and Vascular Normalization**

Mengchao Dong ^1†^, Tingting Zhang ^2†^, Yichun Huang ^3†^, Meng Liu ^1^, Yuhui Yuan ^1^, Bowen Liu ^1^, Qiang Ma ^1^, Haotian Zhang ^1^, Canhua Huang ^2^**,** Chengcheng Shi ^4^*, and Yi Zhang ^1^*

†Mengchao Dong, Tingting Zhang and Yichun Huang contributed equally.

Corresponding author e-mail: [fccshicc@zzu.edu.cn](mailto:fccshicc@zzu.edu.cn); [zhangyi@zzu.edu.cn](mailto:zhangyi@zzu.edu.cn) (leader correspondence);

1. Department of Orthopedic Surgery, The First Affiliated Hospital of Zhengzhou University, Zhengzhou 450052, China
2. Department of Biotherapy Cancer Center and State Key Laboratory of Biotherapy West China Hospital and West China School of Basic Medical Sciences and Forensic Medicine Sichuan University, Chengdu 610041, China
3. The First Affiliated Hospital of Kunming Medical University, Kunming 650031, China
4. Department of Pharmacy, The First Affiliated Hospital of Zhengzhou University, Zhengzhou, China

**Figure S1**. The preparation of D780. (A) The synthetic roadmap of IR780-OH. (B) The ^1^H NMR of IR780-OH (400 MHz, DMSO-*d*_6_, δ). (C) The synthetic roadmap of D780. (D) The HRMS of D780 (APCI) *m/z*.

**Figure S2**. Additional preparation and characterization of OP@NPs. The size distribution of (A) NIB and (B) NIB-Zn. (C) UV-vis absorption spectrum of DPBF at various time intervals following laser irradiation within aqueous solution. Standard curves for (D) NIB and (E) D780. Time-dependent blood concentration curves of (F) NIB and (G) D780 after peritumoral injection of OP@NPs. Each data point represents the mean ± SD of three independent experiments.

**Figure S3**. *In vitro* cytotoxicity and anti-metastatic mechanisms of OP@NPs. Cellular uptake of OP@NPs in K7M2 cells assessed via (A) fluorescence images and (B) flow cytometry analysis. (C) Cellular uptake of D780, NPs, and OP@NPs in K7M2 cells. (D) Flow cytometry data for the cellular uptake of D780, NPs, and OP@NPs in K7M2 cells. Cell viability of (E) MG63 and (F) K7M2 cells after treatment with NIB-Zn, D780, NPs, and OP@NPs without laser irradiation (n = 3, Two-way ANOVA). (G) Images and (H) statistical analysis of colony formation in K7M2 cells after treatment with NIB, NIB-Zn, D780, NPs, and OP@NPs under laser irradiation (n = 3, 1.0 W·cm⁻², 660 nm, 30 s, Student’s *t*-test). (I) Fluorescent images and (J) statistical analysis of EdU labeling assay in K7M2 cells under different conditions (n = 3, Student’s *t*-test). (K) Representative fluorescent images and (L) statistical analysis of DCFH-DA staining in K7M2 cells under different conditions (n = 3, Student’s *t*-test). (M-N) Flow cytometry analysis of DCFH-DA in K7M2 cells under different conditions (n = 3, Student’s *t*-test). (O) Fluorescent images and (P) analysis of JC-1 staining in K7M2 cells under different conditions (n = 3, Student’s *t*-test). (Q) Live/Dead staining and (R) analysis of K7M2 cells under different conditions (n = 3, Student’s *t*-test). (S-T) JC-1 flow cytometry analysis and (U-V) apoptosis flow cytometry analysis of K7M2 cells under different conditions (n = 3, Student’s *t*-test). (W) Wound healing assay for migration evaluation of K7M2 cells. (X) Migration and (Y) invasion detected by Transwell assays in K7M2 cells. Quantitative analysis of (Aa) wound healing, (Ab) migration, and (Ac) invasion (n = 3, Student’s *t*-test). Detection of (Z) EMT-related proteins and (Ad) VEGF expression in K7M2 cells (n = 3). Each data point represents the mean ± SD of three independent experiments. (* *P* < 0.05; ** *P* < 0.01; *** *P* < 0.001).

**Figure S4**. Metabolic reprogramming of glycolysis by OP@NPs. The low lactate metabolism group is represented by Cluster 1, while the high lactic acid metabolism group is represented by Cluster 2. (A) The ratio of immune cells in the two clusters. (B) LA, (C) ATP, and (D) ECAR assays of K7M2 cells after treatment with NIB, NIB-Zn, D780, NPs, and OP@NPs under laser irradiation (n = 3, 1.0 W·cm⁻², 660 nm, 30 s, Student’s *t*-test). (E) Immunoblot analysis of lactate metabolism-related proteins in K7M2 cells (n = 3). (F-G) Flow cytometry analysis, (H) representative fluorescent images and (I) analysis of 2-NBDG staining in K7M2 cells (n = 3, Student’s *t*-test). (J) Immunoblot analysis of GLUT1 expression (n = 3). (K) Live/dead staining of K7M2 cells and (L) analysis with or without pyruvate supplementation (n = 3, Student’s *t*-test). (M) LA inhibition rate in K7M2 cells with or without pyruvate supplementation (n = 3, Student’s *t*-test). Each data point represents the mean ± SD of three independent experiments. (* *P* < 0.05; ** *P* < 0.01; *** *P* < 0.001).

**Figure S5**. Vascular remodeling and immune activation by OP@NPs. (A) Schematic illustration of the Transwell co-culture system. (B) TGF-β, IL-2, TNF-α, IFN-γ, GZM B and PRF1 expression in the culture medium detected by ELISA (n = 3). Flow cytometry results of (C-D) Tregs (CD4⁺CD25⁺Foxp3⁺), (E-F) M1 TAMs (CD11b⁺iNOS⁺F4/80⁺), and (G-H) NK cells (CD3^-^CD11b^+^NK-L1^+^) in the spleen, (I-J) DCs (CD11c^+^CD80^+^CD86^+^), (K-N) M1 TAMs (CD11b^+^iNOS^+^F4/80^+^), and M2 TAMs (CD11b^+^CD206^+^F4/80^+^) in the bone marrow (n = 3, Student’s *t-*test). Each data point represents the mean ± SD of three independent experiments. (* *P* < 0.05; ** *P* < 0.01; *** *P* < 0.001).

**Figure S6.** Anti-tumor immunity activation and biosafety of OP@NPs *in vivo*. Immunohistochemical TNF-α analysis in (A) DCs and (B) macrophages (n = 3, Student’s *t*-test). Flow cytometry results of (C-D) M1 TAMs (CD11b+iNOS+F4/80+) and (E-F) M2 TAMs (CD11b+CD206+F4/80+) following indicated treatments in tumor (n = 3, Student’s *t*-test). Flow cytometry results of (G-I) T cells (CD3⁺CD4⁺/CD3⁺CD8⁺), (J-K) Tregs (CD4⁺CD25⁺Foxp3⁺), (L-M) DCs (CD11c⁺CD80⁺CD86⁺), (N-O) M1 TAMs (CD11b⁺iNOS⁺F4/80⁺) and (P-Q) M2 TAMs (CD11b⁺CD206⁺F4/80⁺) after indicated treatments in the spleen (n = 3, Student’s *t*-test). (R) H&E staining images of major organs from each treatment group (n = 3). (S) Hemolysis test of OP@NPs with different concentrations (n = 3). (T-V) Blood biochemical markers: (T) AST, (U) CRE, (V) URE (n = 3, Student’s *t*-test). Each data point represents the mean ± SD. (** *P* < 0.01; *** *P* < 0.001).

**Figure S7.** Vascular normalization drives antitumor immune activation *in vivo*. (A) Tumor inhibition efficiency of indicated groups (n = 5, Student’s *t*-test). (B) Body weight of mice from different treatment groups at different time points (n = 5). Flow cytometry analysis and quantification of (C-D) CD3⁺CD4⁺ T cells and (E-F) iNOS⁺ M1 macrophages in tumor. Each data point represents the mean ± SD. (* *P* < 0.05; ** *P* < 0.01; *** *P* < 0.001).

**Figure S8.** OP@NPs enhance sensitization to PD-1 antagonist pembrolizumab in osteosarcoma treatment. (A) Schematic illustration of *in vivo* combined PD-1 inhibitor therapy. (B) Tumor growth curves of mice in each group (n = 5, Student’s t-test). (C) Representative photographs of tumors (n = 5). (D) Tumor inhibition rates. (E) PD-L1 expression in tumor tissues. (F-G) Immunohistochemical staining and quantification of PD-1 in tumor tissues (n = 3, Student’s t-test). (H-K) Flow cytometric detection of PD-1 and PD-L1 in tumor tissues (n = 3). (L-N) Flow cytometry analysis of CD3⁺CD4⁺ and CD3⁺CD8⁺ T cells in tumor tissues (n = 3). (O-Q) Flow cytometry analysis of CD3⁺CD4⁺ and CD3⁺CD8⁺ T cells in spleens (n = 3, Student’s t-test). Each data point represents the mean ± SD. (* P < 0.05; ** P < 0.01; *** P < 0.001).

**Table S1.** Flow cytometry antibodies in this study.

| **Antibody name** | **Company and Cat. No.** |
| --- | --- |
| FITC anti-mouse CD3 Antibody | Biolegend, 100203 |
| APC anti-mouse CD4 Antibody | Biolegend, 100412 |
| PE anti-mouse CD8a Antibody | Biolegend, 100708 |
| FITC anti-mouse CD80 Antibody | Biolegend, 104705 |
| APC anti-mouse CD86 Antibody | Biolegend, 159215 |
| PE anti-mouse CD11c Antibody | Biolegend, 117307 |
| FITC anti-mouse F4/80 Antibody | Biolegend, 157310 |
| PE anti-iNOS Antibody | Biolegend, 696806 |
| PerCP/Cyanine5.5 anti-mouse CD206 Antibody | Biolegend, 141716 |
| FITC anti-mouse CD25 Antibody | Biolegend, 102006 |
| PE anti-mouse FOXP3 Antibody | Biolegend, 126404 |
| PerCP anti-mouse/human CD11b Antibody | Biolegend, 101229 |
| PE anti-mouse/human CD44 Antibody | Biolegend, 103007 |
| PerCP anti-mouse CD62L Antibody | Biolegend, 104430 |

**Table S2.** Primary antibodies in this study.

| **Antibody name** | **Company and Cat. No.** |
| --- | --- |
| β-actin | Cell Signaling Technology, #4967 |
| LDHA | Cell Signaling Technology, #2012 |
| HIF-1α | Cell Signaling Technology, #48085 |
| MCT4 | Cell Signaling Technology, #81569 |
| c-MYC | Abcam, #ab185656 |
| HKII | Cell Signaling Technology, #2867 |
| GLUT1 | Cell Signaling Technology, #73015 |
| VEGF | ABclonal, #A23759 |
| PD-L1 | ABclonal, #A19135 |
| E-cadherin | ABclonal, #A20798 |
| Vimentin | ABclonal, #A19607 |
| TNF-α | ABclonal, #A11534 |
| CD11c | Cell Signaling Technology, #97585 |
| F4/80 | Cell Signaling Technology, #71299 |
| Ki67 | Cell Signaling Technology, #9449 |
| TUNEL | Cell Signaling Technology, #25879 |

**Table S3.** Assay Kits in this study.

| **Antibody name** | **Company and Cat. No.** |
| --- | --- |
| Reactive Oxygen Species Assay Kit | Beyotime, S0033S |
| Annexin V-FITC/PI Apoptosis Detection Kit | BD, #556547 |
| Mitochondrial membrane potential assay kit with JC-1 | Beyotime, C2006 |
| ATP Assay Kit | Beyotime, S0026 |
| LA Assay Kit | Solarbio, #BC2235 |
| ECAR Assay Kit | Elabscience, #E-BC-F069 |
| 2-NBDG Assay Kit | Beyotime, #S0561S |
| Mouse TGF-β ELISA Kit | BOSTER, #EK0515 |
| Mouse IL-2 ELISA Kit | BOSTER, #EK0398 |
| Mouse TNF-α ELISA Kit | BOSTER, #EK0527 |
| Mouse PRF1 ELISA Kit | Elabscience, # E-EL-M0890 |
| Mouse IFN-γ ELISA Kit | BOSTER, #K0375 |
| Mouse GZM B ELISA Kit | BOSTER, #EK1115 |

**Table S4.** Chemical reagents in this study.

| **Antibody name** | **Company and Cat. No.** |
| --- | --- |
| Sodium alginate | Aladdin Reagent Co., Ltd. S100127 |
| Sodium periodate | Aladdin Reagent Co., Ltd. S104093 |
| Ethylene glycol | Aladdin Reagent Co., Ltd. E119700 |
| IR-780 iodide | Aladdin Reagent Co., Ltd. I302749 |
| 3-Amino-1-propanol | Aladdin Reagent Co., Ltd. A106069 |
| Ethylene imine polymer (PEI) | Aladdin Reagent Co., Ltd.P434393 |
| Diclofenac Sodium | Aladdin Reagent Co., Ltd. D129332 |
| BIBF 1120 | Aladdin Reagent Co., Ltd. N129725 |
| Anti-Mouse VEGFR-2 Antibody | MedChemExpress, #  HY-P990106 |
| Pyruvic acid | Aladdin Reagent Co., Ltd. P421121 |
| Zn(NO)_3·_H_2_O | Aladdin Reagent Co., Ltd. Z118841 |
| EDCI | Aladdin Reagent Co., Ltd. E106172 |
| Triethanolamine (TEA) | Aladdin Reagent Co., Ltd.T431269 |
| NHS-PEG-OH | Aladdin Reagent Co., Ltd.N164052 |
| 4-Dimethylaminopyridine | Aladdin Reagent Co., Ltd.109207 |
